# Polypharmacology of Pathway Crosstalk in Neurodegenerative Diseases: Chemical Modulation of Interconnected Signaling Networks

**DOI:** 10.3390/cells15110962

**Published:** 2026-05-22

**Authors:** Muhammad Sohail Khan, Imran Zafar, Muhammad Noman, Gabsik Yang, Ki Sung Kang, Jean C. Bopassa

**Affiliations:** 1 College of Korean Medicine, Gachon University, 1342 Seongnamdaero, Seongnam 13120, Republic of Korea; 2 Department of Cellular and Integrative Physiology, School of Medicine, University of Texas, Health Science Center at San Antonio (UTHSCSA), 7703 Floyd Curl Dr., San Antonio, TX 78229, USA; bopassa@uthscsa.edu; 3 Department of Biochemistry and Biotechnology, Faculty of Science, The University of Faisalabad (TUF), Faisalabad 38000, Punjab, Pakistan; bioinfo.pk@gmail.com; 4 Department of Medical Laboratory Sciences, Faculty of Allied Health Sciences and Medicine, The University of Faisalabad (TUF), Faisalabad 38000, Punjab, Pakistan; muhammadnoman.mls1@gmail.com

**Keywords:** polypharmacology, pathway crosstalk, neurodegeneration, network pharmacology, multi-target drugs, systems biology

## Abstract

Neurodegenerative disorders, including Alzheimer’s disease (AD), Parkinson’s disease (PD), Huntington’s disease (HD), and amyotrophic lateral sclerosis (ALS), arise from highly interconnected molecular and cellular abnormalities that progressively lead to neuronal dysfunction, synaptic failure, and cell death. This review provides a unified framework to understand the interrelated molecular mechanisms driving these diseases, with a focus on identifying key disease-specific intervention nodes. Core contributors include oxidative stress, mitochondrial dysfunction, protein aggregation, neuroinflammation, and emerging roles of peroxisomal dysfunction in redox imbalance, lipid dysregulation, and inflammatory amplification. Single-target therapies often show limited efficacy due to the complex, interconnected nature of these pathways. In contrast, polypharmacology, which targets multiple disease-relevant mechanisms simultaneously, offers a more promising therapeutic strategy. This review critically examines how pathway crosstalk drives neurodegenerative progression, with particular emphasis on mitochondrial–ROS–inflammatory signaling, aggregation–proteostasis failure, synaptic–neuroimmune dysfunction, and gut–brain communication. It evaluates various multi-node intervention strategies, including multi-target-directed ligands (MTDLs), molecular hybrids, natural products, drug repurposing, and nanocarrier-based delivery systems. Advances in network pharmacology, artificial intelligence (AI), bioinformatics, and multi-omics have enhanced the identification of actionable therapeutic nodes, candidate compounds, and brain-targeted delivery platforms. Notably, the NOD-like receptor pyrin domain-containing protein 3 (NLRP3) inflammasome and cyclic GMP–AMP synthase (cGAS)—stimulator of interferon genes (STING) pathways—play distinct roles in neuroinflammation, amplifying neuronal damage by releasing inflammatory cytokines and inducing mitochondrial dysfunction. However, successful translation into clinical practice remains constrained by challenges such as blood–brain barrier penetration, patient heterogeneity, and biomarker limitations. The review advocates for a shift towards mechanism-informed, patient-stratified polypharmacological strategies to better address the network pathology of neurodegeneration, despite significant translational hurdles.

## 1. Introduction

Neurodegenerative diseases (NDs), including Alzheimer’s disease (AD), Parkinson’s disease (PD), Huntington’s disease (HD), and amyotrophic lateral sclerosis (ALS), are a growing global health burden characterized by progressive neuronal loss that leads to severe cognitive and motor impairments [1]. These disorders significantly reduce patients’ quality of life and impose substantial socioeconomic burdens on healthcare systems worldwide [2]. However, effective disease-modifying therapies remain limited because these conditions are complex and multifactorial, arising from dynamically interacting biological processes rather than a single molecular defect [3]. Oxidative stress and mitochondrial dysfunction are critical in the pathogenesis of NDs [4]. Peroxisomal dysfunction is also increasingly recognized as an important contributor to neuronal vulnerability, regulating redox balance, lipid metabolism, plasmalogen biosynthesis, and inflammatory signaling [5,6]. Dysfunction in peroxisomes can exacerbate mitochondrial stress, amplify neuroinflammation, and disrupt neuronal survival [7].

The blood–brain barrier (BBB) is one of the greatest impediments to effective treatment, limiting the penetration of therapeutic agents into the central nervous system and the drugs’ effects [8]. To overcome this difficulty, new drug delivery methods, such as nanotechnology-based systems, have been investigated to improve brain targeting and pharmacokinetics [9]. Moreover, new therapeutic modalities, including selective protein degradation technologies, gene therapy, and neurotrophic factor-based therapies, have demonstrated potential for treating the disease’s pathology at multiple levels. Nevertheless, their use in the clinic is still constrained by delivery efficiency, invasiveness, and long-term safety concerns [10].

**Figure 1:**
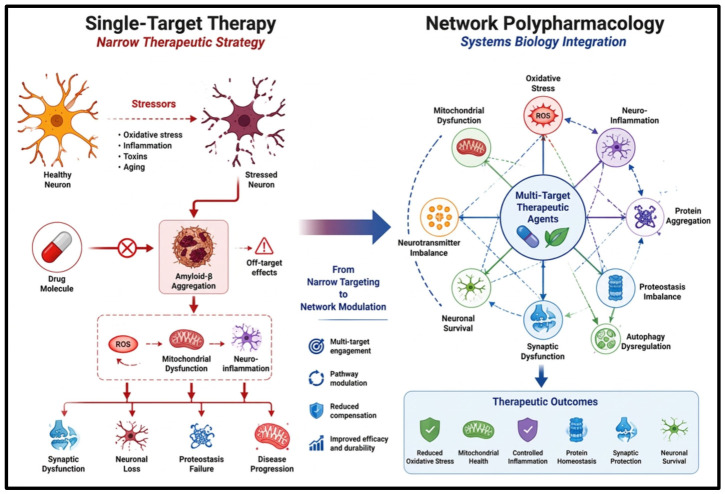
From single-target therapy to network-based polypharmacology in NDs.

Traditionally, drug discovery has been driven by the one-drug, one-target paradigm, aiming to regulate a single molecular target selectively. Although this method has been effective for some diseases, it has not been effective for NDs. The failures of therapies based on the reductionist approach, which aim to isolate mechanisms such as amyloid-β in AD or dopamine pathways in PD, demonstrate the inefficacy of this approach [11]. Such weaknesses are mostly explained by compensatory biological processes, pathway redundancy, and the activation of other signaling pathways that enable disease development despite specific interventions [12]. Figure 1 shows a conceptual weakness by comparing the conventional single-target paradigm with a network-based polypharmacological model.

NDs are now being viewed as network-based diseases with complex interactions between many dysregulated pathways. Pathological processes such as oxidative stress, mitochondrial dysfunction, neuroinflammation, and protein aggregation are closely interlinked and contribute to neuronal damage. These processes are not independent; they are interconnected by complex signaling networks and feedback loops that enhance disease progression [13]. To demonstrate, inflammatory signaling may be triggered by oxidative stress, which, in turn, impairs mitochondrial function and promotes protein aggregate formation, creating a self-reinforcing loop of neuronal damage [14].

Recent progress in systems biology has provided insight into these interactions and how they combine to create disease-related networks, leveraging multi-omics data and computational models [15]. The pathophysiology of AD, PD, HD, and ALS shares common pathways, including oxidative stress and neuroinflammation [16]. However, each disease is also characterized by unique molecular triggers, such as amyloid-β in AD and α-synuclein in PD. Earlier studies highlight distinctions and their relevance for polypharmacological strategies [17]. The holistic methodology allows the identification of convergent pathways and key regulatory nodes that can serve as potential targets for therapeutic interventions [18]. Additionally, connectome-related work has shown the dissemination of pathological proteins within neural networks, underscoring the importance of system-level insights for the development of precision medicine approaches [19].

Pathway crosstalk has been central to neurodegeneration, as it allows different signaling cascades to communicate with one another and is involved in neuronal survival and neuronal death [20]. The connections among oxidative stress, inflammatory processes, mitochondrial dysfunction, and apoptotic signaling form an integrated network that regulates disease progression. Moreover, there is cross-talk between key regulatory pathways, e.g., Hippo and Wnt signaling, that extends to inter-organelle communication, e.g., mitochondria-lysosome interactions, which are needed to sustain cellular homeostasis [21]. It is also further outlined in the interplay between apoptosis and autophagy, as well as in DNA repair mechanisms, which makes these systems more complex [22]. Knowledge of these interactions is crucial for identifying major network hubs that can be targeted for therapy.

Polypharmacology has become an effective new form of therapy in response to these difficulties, which seeks to adjust the various targets at the same time. Polypharmacology refers to the design of therapies that simultaneously target multiple disease-related pathways. Multi-target-directed ligands (MTDLs) are single molecules designed to engage several targets at once, whereas network pharmacology is a broader approach that maps the interactions between drugs and disease networks to identify actionable therapeutic nodes [23]. In contrast to classical single-target-oriented methods, the concept of polypharmacology aims to design MTDLs that interact with multiple pathways associated with the disease and can thus address the complexity of NDs more effectively [24]. Such an approach can optimize therapeutic benefit, minimize the risk of resistance, and potentially improve clinical outcomes by addressing interconnected signaling pathways rather than single molecular components [24].

The latest advances in medicinal chemistry have enabled the development of multi-target agents using techniques such as molecular hybridization, in which the pharmacophoric components of various bioactive molecules are incorporated into a single molecule [25]. Synthetic compounds, as well as natural products such as polyphenols and alkaloids, have been shown to regulate signaling pathways implicated in neurodegeneration, including the JAK/STAT and PI3K/Akt pathways [26]. Moreover, computational methods, such as molecular docking, quantitative structure–activity relationship modeling, and artificial intelligence (AI), have played a crucial role in accelerating the identification and optimization of multi-target drug candidates [27].

Computational In-silico methods have also improved drug discovery by enhancing efficiency through high-throughput screening technologies that enable rapid screening of large compound libraries, thereby increasing the probability of discovering a clinically viable compound [28]. Besides this, network pharmacology is a combination of systems biology and pharmacology that offers an all-encompassing methodology for capturing drug-target interactions within intricate biological systems to support the rational design of polypharmacological therapies [12]. All these innovations demonstrate the need to shift to systems-level approaches and reduce dependence on reductionist approaches to better manage the complexity of NDs.

This review addresses a specific gap in the neurodegeneration literature by moving beyond descriptive summaries of individual pathological mechanisms and focusing on how these mechanisms converge into therapeutically actionable network nodes. The primary objective of this review is to critically evaluate how pathway crosstalk contributes to neurodegenerative progression and how polypharmacological strategies can be used to modulate multiple disease-relevant mechanisms in a rational, disease-informed manner. Although neuroinflammation, mitochondrial dysfunction, protein aggregation, impaired proteostasis, synaptic failure, and metabolic dysregulation have often been reviewed separately, their functional interdependence is central to disease progression and therapeutic resistance. Therefore, this review is organized around four convergent axes of pathway crosstalk: mitochondrial–ROS–inflammatory signaling, protein aggregation–proteostasis failure, synaptic–neuroimmune dysfunction, and computational/network-guided multi-target drug discovery. Within this framework, endocannabinoid modulation, natural products, nanomedicine, AI, and drug repurposing are discussed as representative polypharmacological strategies rather than as independent review themes. The central argument is that effective therapeutic development for NDs requires rational modulation of disease-relevant network nodes while accounting for disease-specific biology, blood–brain barrier limitations, safety, patient heterogeneity, biomarker stratification, and clinical translatability.

## 2. Literature Search Strategy

This review was conducted as a structured narrative review to synthesize mechanistic, pharmacological, computational, and translational evidence on polypharmacology and pathway crosstalk in NDs. A narrative approach was chosen to integrate heterogeneous molecular, experimental, computational, and clinical evidence across diverse therapeutic and mechanistic domains [29,30,31]. Relevant literature was identified through searches of *PubMed/MEDLINE*, *Web of Science*, *Scopus*, and *Google Scholar*. Search terms combined with Boolean operators included *“neurodegenerative diseases,” “Alzheimer’s disease,” “Parkinson’s disease,” “Huntington’s disease,” “amyotrophic lateral sclerosis,” “polypharmacology,” “multi-target-directed ligands (MTDLs),” “network pharmacology,” “mitochondrial dysfunction,” “oxidative stress,” “reactive oxygen species (ROS),” “neuroinflammation,” “NF-κB,” “NLRP3 inflammasome,” “cGAS–STING,” “proteostasis,” “autophagy,” “lysosomal dysfunction,” “endocannabinoid system,” “drug repurposing,” “artificial intelligence,” “machine learning,” “nanomedicine,” “blood–brain barrier,” “peroxisome,” “peroxisomal dysfunction,” “pexophagy,” “plasmalogen,” “very-long-chain fatty acids (VLCFAs),”* and *“peroxisome–mitochondria crosstalk”.* The search focused on peer-reviewed studies from the last 10 years, with key older studies included when necessary. Priority was given to mechanistic, translational, clinical, and high-quality review articles. Studies addressing major disease pathways and neurodegenerative disorders were included. Research on multi-target and computational therapeutic strategies was also considered. This article is a critical narrative synthesis rather than a systematic review; formal PRISMA-based screening, risk-of-bias scoring, and pooled statistical analysis were not performed. However, the reporting of the literature-search process was guided by principles of transparency, methodological clarity, and reproducible review conduct, as recommended for high-quality narrative and evidence synthesis articles [30,31,32,33]. The final synthesis emphasized conceptual relevance, mechanistic depth, translational importance, and the strengths and limitations of available evidence.

## 3. Molecular Architecture of Neurodegeneration

NDs are a heterogeneous group of diseases characterized by the progressive deterioration of neuronal structure and function, which eventually leads to cognitive impairment, motor, and behavioral deviations. The molecular characteristics of these diseases include the deposition of misfolded, aggregation-prone proteins such as amyloid-β (Aβ), tau, α-synuclein, and TDP-43. These proteins form highly organized amyloid fibrils that disrupt cell homeostasis, leading to specific pathological diseases [34]. There is growing evidence that the propagation of these aggregates occurs through prion-like processes, which involve the use of templates for misfolding and intercellular transfer, thereby contributing to disease development and heterogeneity.

New capabilities of cryo-electron microscopy have enabled high-resolution characterization of disease-specific amyloid structures, providing key insights into the aggregation process and helping develop targeted therapeutic strategies [35]. Protein aggregation, however, is not a sufficient explanation of neurodegeneration. Instead, it is a part of a bigger system of interrelated molecular processes, including oxidative stress, dysfunction in the mitochondrion, neuroinflammation, improper proteostasis, and genetic susceptibility factors [36]. These processes are further enhanced by aging, which leads to genomic instability, cellular senescence, and reduced repair potential, therefore rendering neurons more susceptible. As a result, new therapeutic approaches are not based on symptomatic treatment but on disease-modifying methods, such as gene editing, nanotechnology-based drug delivery, and stem cell therapies [37].

### 3.1. Protein Aggregation and Cross-Seeding

Protein aggregation is a key, convergent characteristic of NDs, and specific proteins are associated with specific disorders. The aggregates of Amyloid-β and tau are typical in AD, and the aggregates of α-synuclein in the Lewy bodies of PD and other synucleinopathies. Notably, such aggregation processes do not occur in a vacuum but rather interact with one another through complex cross-seeding, which accelerates disease progression [38]. Molecularly, aggregation begins with protein misfolding, followed by soluble oligomers and insoluble fibrillar structures. These toxic intermediates interfere with cellular proteostasis systems, including molecular chaperones, ubiquitin, the proteasome system, autophagy pathways, and the unfolded protein response. Toxicity is further enhanced by cross-talk of aggregation-prone proteins. For example, amyloid-β can mediate the aggregation of α-synuclein via heterogeneous nucleation, and the co-aggregation of tau and α-synuclein via electrostatic and post-translational interactions [39]. Another level of complexity is liquid–liquid phase separation (LLPS), which promotes the formation of dynamic protein condensates that can transform into irreversible amyloid aggregates in pathological conditions [40]. Additional imaging and fluorescence-based methods have also provided kinetic data on the initial aggregation events, enabling the discovery of pre-fibrillar toxic species. These results, together with previous studies, underscore the dynamic and interconnected nature of protein aggregation, and thus, multi-target therapeutic approaches that can inhibit aggregation, disrupt cross-seeding, and promote protein clearance are desirable.

### 3.2. Oxidative Stress, Mitochondrial Dysfunction, and Neuroinflammation

**Figure 2:**
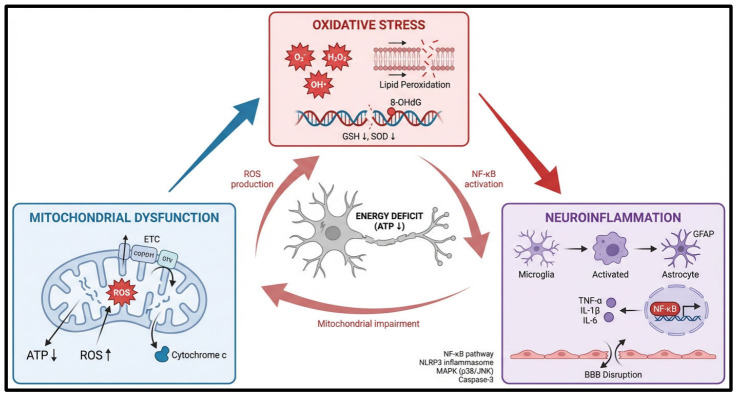
Interconnected Cellular Mechanisms of Oxidative Stress, Neuroinflammation, and Mitochondrial Dysfunction.

Neurodegenerative diseases are driven by the interconnected triad of oxidative stress, mitochondrial dysfunction, and neuroinflammation, where impaired mitochondrial respiration, calcium dysregulation, defective mitophagy, and excessive ROS production lead to oxidative damage and progressive neuronal degeneration [41,42]. Peroxisomal dysfunction further contributes to ROS accumulation, lipid peroxidation, impaired β-oxidation, and reduced plasmalogen synthesis, thereby amplifying oxidative stress and inflammatory signaling in neurons [43]. Damaged mitochondria release mtDNA and other mitochondrial DAMPs that activate the cGAS–STING and NF-κB pathways [42], while mitochondrial ROS and oxidized mtDNA stimulate NLRP3 inflammasome activation and the maturation of IL-1β and IL-18 [44]. Although cGAS–STING and NLRP3 signaling may converge, cGAS–STING primarily mediates cytosolic DNA sensing and interferon signaling, whereas NLRP3 regulates inflammasome-dependent cytokine activation. Chronic activation of microglia and astrocytes further releases TNF-α, IL-1β, IL-6, nitric oxide, and ROS, creating a feed-forward cycle that worsens mitochondrial dysfunction, synaptic impairment, and neuronal death in disorders such as Alzheimer’s disease, Parkinson’s disease, ALS, and Huntington’s disease [44,45]. Consequently, therapies targeting mitochondrial ROS, mitophagy, inflammasomes, and STING signaling may help restore redox and immune homeostasis rather than completely suppressing inflammation [46]. Figure 2 illustrates this vicious cycle linking mitochondrial dysfunction, ROS generation, inflammatory signaling, and neurodegeneration.

### 3.3. Autophagy–Lysosomal Failure and Proteostasis Collapse

**Figure 3:**
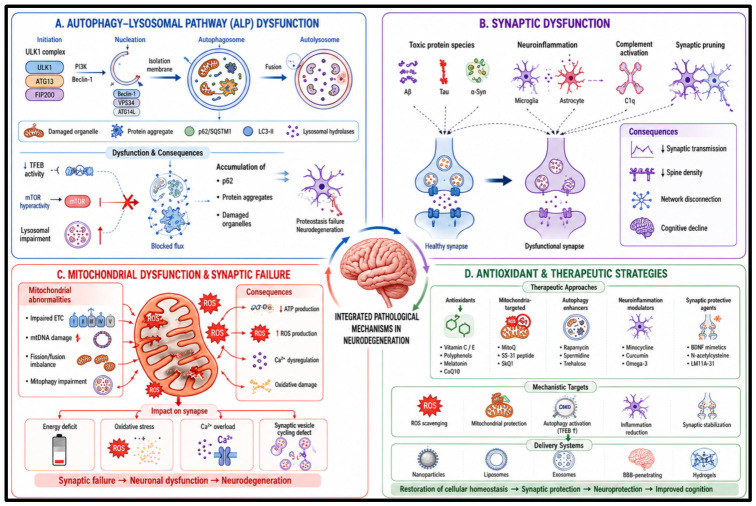
Integrated pathological mechanisms in neurodegeneration and therapeutic strategies. (**A**) Autophagy–lysosomal pathway dysfunction leading to impaired proteostasis and accumulation of toxic protein aggregates. (**B**) Synaptic dysfunction driven by toxic protein species, neuroinflammation, and synaptic pruning. (**C**) Mitochondrial dysfunction contributes to synaptic failure through energy deficits, oxidative stress, and calcium dysregulation—(**D**) Antioxidant and mitochondria-targeted therapeutic strategies aimed at restoring cellular homeostasis and mitigating neurodegenerative pathology.

This impairment results in the accumulation of pathogenic proteins such as amyloid-β and hyperphosphorylated tau, which further disrupt lysosomal integrity and autophagic flux. Consequently, a vicious cycle is established in which protein aggregation and autophagic dysfunction mutually reinforce one another, exacerbating cellular stress and neuronal damage [47]. Increased autophagosome number may reflect either enhanced autophagy initiation or impaired lysosomal clearance [48]. Key regulatory points include mTOR-dependent autophagy initiation, AMPK activation, regulation of the ULK1 complex, TFEB-mediated lysosomal biogenesis, lysosomal acidification, and cargo recognition via receptors such as p62/SQSTM1 [49]. Therapeutic enhancement of autophagy is therefore context-dependent: stimulating autophagosome formation without restoring lysosomal degradation may worsen intracellular accumulation rather than improve proteostasis [50]. Genetic predisposition is a major cause of ALP dysfunction. Genetic susceptibility is associated with mutations of genes that interrupt endo-lysosomal trafficking, protein sorting, and autophagic pathways: PSEN1/2, APOE4, SORL1, and TREM2 [51]. The therapeutic interventions based on ALP, such as TFEB activation and mTOR modulation, are designed to restore autophagic flux and lysosomal activity, as shown in Figure 3A (human-like neuron with dysfunctional autophagosomes and lysosomes filled with protein aggregates). Still, the key element to consider here is maintaining fine control to prevent undesirable effects [52].

### 3.4. Synaptic Dysfunction and Network Vulnerability

Synaptic dysfunction is an early and vital phenomenon in neurodegeneration and is more significantly linked with cognitive impairment than traditional pathological markers. This is caused by toxic protein oligomers, especially amyloid-β oligomers, which interfere with normal synaptic transmission and glutamatergic signaling via NMDA and AMPA receptors, leading to calcium imbalances and excitotoxicity [53]. Microtubule destabilization, axonal transport impairment, and disruption of synaptic protein trafficking are also caused by tau pathology [54]. Simultaneously, neuroinflammation increases synaptic damage via microglial-mediated synaptic pruning and astrocytic impairment, thereby impairing glutamate clearance and increasing excitotoxic stress [55]. At the structural level, synaptic dysfunction is characterized by the loss of dendritic spines, decreased synaptic density, and altered network connectivity, which eventually result in cognitive and behavioral deficits [56]. These features are emphasized in Figure 3B, which shows semi-realistic loss of dendritic spines, disruption of synaptic clefts, and microglial loss of synapses. The treatment modalities aim to restore synaptic plasticity, regulate neurotransmitter systems, and mitigate protein toxicity and inflammation.

### 3.5. Mitochondrial Dysfunction and Synaptic Failure

Mitochondrial dysfunction is critical to synaptic failure because synaptic activity is energetically intensive. Mitochondria control ATP production, calcium homeostasis, and redox homeostasis, which are required for neurotransmission and synaptic plasticity [57]. Dysfunctional mitochondria reduce mitochondrial activity, synaptic vesicle recycling, and calcium buffering, leading to synaptic degeneration and excitotoxicity [58]. Mitochondrial dynamic changes, such as faulty fission, fusion, transport, and mitophagy, lead to the development of pathological mitochondria in synapses. Disease-associated proteins, including amyloid-β, tau, and α-synuclein, further impair mitochondrial function, thereby accelerating synaptic degeneration [59]. Figure 3C demonstrates mitochondrial fragmentation, ROS overproduction, impaired ATP production, and defective calcium buffering in synaptic terminals, underscoring the correlation between mitochondrial dysfunction and synapse degeneration. These findings emphasize mitochondria as an essential therapeutic target in NDs.

### 3.6. Antioxidant Therapies in Neurodegenerative Diseases

Antioxidant therapies are promising interventions for reducing oxidative stress and its consequences in NDs. These include enzymatic antioxidants such as superoxide dismutases and non-enzymatic antioxidants, including vitamins C and E, glutathione, polyphenols, and melatonin [60]. Mitochondria-targeted antioxidants, such as MitoQ and SS-31, exhibit enhanced activity by directly suppressing mitochondrial ROS production and improving bioenergetic function. Nevertheless, it is not easily translated to the clinic due to low bioavailability, low BBB permeability, and potential toxicity [61]. To address these shortcomings, the focus is on nanoparticle-based delivery systems and combination therapies targeting multiple pathological pathways. The impact of these antioxidant and multi-target interventions is illustrated in Figure 3D, which shows ROS scavenging, mitochondrial recovery, autophagy reactivation, and reduced neuroinflammation in human-like neuronal models. Although the preclinical findings are encouraging, they need to be validated in strong clinical studies [62].

## 4. Pathway Crosstalk as a Driver of Neurodegenerative Progression

### 4.1. Network-Based View of Neurodegeneration

NDs are no longer viewed as the consequence of isolated molecular abnormalities but rather as the outcome of progressive disruption within an interconnected cellular network. Neuronal survival depends on the coordinated function of multiple systems, including redox regulation, mitochondrial bioenergetics, proteostasis, immune signaling, and synaptic communication. Disturbance in any one of these systems rapidly propagates across others through tightly coupled biochemical interactions, ultimately leading to widespread cellular dysfunction and neuronal loss [62]. A key feature of this network behavior is the shift from adaptive to maladaptive responses. Under physiological conditions, pathways such as oxidative stress responses and inflammation serve protective roles by maintaining cellular homeostasis. However, chronic or excessive activation transforms these mechanisms into drivers of pathology. Persistent oxidative stress promotes protein misfolding and mitochondrial damage, while sustained inflammation disrupts neuronal signaling and survival pathways. A more actionable interpretation of this crosstalk is that some molecules operate as network convergence points. For example, mitochondrial damage can activate ROS-dependent NF-κB signaling, NLRP3 inflammasome activation, and mtDNA-driven cGAS–STING responses. These pathways do not simply coexist; they convert organelle stress into inflammatory signaling and may determine whether cells undergo adaptive repair, chronic inflammation, or death. Such convergence nodes are particularly relevant to polypharmacology because they provide rational points at which multi-target intervention may interrupt feed-forward degeneration [63]. Importantly, this systems-level perspective introduces the concept of a critical threshold in disease progression. Early compensatory mechanisms may temporarily maintain cellular function, but once a tipping point is reached, interconnected pathological pathways become self-sustaining. At this stage, neurodegeneration progresses independently of the initial trigger, highlighting the limitations of single-target therapeutic approaches and emphasizing the need for multi-pathway interventions [64].

### 4.2. The Mitochondria–ROS–Inflammation Axis

Mitochondrial dysfunction, oxidative stress, and inflammation are some of the main pathogenic axes of interaction in NDs. The role of mitochondria in energy production is critical; they also regulate redox and innate immune signaling. When mitochondrial function is disrupted, excess ROS are produced due to inefficiency of the electron transport chain, the cellular antioxidant systems are overburdened, and molecular damage begins [65]. ROS are signaling molecules that stimulate inflammatory responses, such as activation of NF-κB and the NLRP3 inflammasome, leading to the release of pro-inflammatory cytokines. These cytokines, in turn, trigger mitochondrial dysfunction and amplify ROS generation into a feed-forward loop that can perpetuate cell damage [66]. In addition to their roles in oxidative stress and inflammation, mitochondria play a crucial role in controlling cell fate. Mitochondrial membrane leakage triggers cytochrome c release and caspase-dependent apoptosis, linking metabolic dysfunction to programmed cell death. Moreover, mitochondrial calcium dysregulation leads to excitotoxicity and synaptic instability. Collectively, these mechanisms render mitochondria a key point of intersection where a variety of neurodegenerative mechanisms collide [63].

The therapeutic implication of the mitochondria–ROS–inflammation axis is not simply that these processes coexist, but that mitochondrial injury can convert metabolic stress into innate immune activation [67]. This makes mitochondrial quality control, mtDNA release, NLRP3 inflammasome activation, cGAS–STING signaling, and redox-sensitive NF-κB activation actionable convergence nodes [68]. However, these nodes are biologically context-dependent: transient inflammatory signaling may support repair and debris clearance, whereas chronic activation promotes cytokine release, synaptic dysfunction, and neuronal death. Therefore, therapeutic strategies should aim to restore homeostatic signaling rather than completely suppress inflammatory or redox responses.

### 4.3. The Peroxisome–ROS–Inflammation Axis

Peroxisomes represent an additional redox-sensitive organelle system involved in the progression of neurodegenerative diseases (NDs). Although mitochondria are considered the major source of neuronal reactive oxygen species (ROS), peroxisomes also regulate ROS metabolism through oxidase-dependent hydrogen peroxide production and catalase-mediated detoxification [69]. Impaired peroxisomal quality control may lead to the accumulation of damaged peroxisomes, sustained oxidative stress, disrupted membrane lipid composition, synaptic dysfunction, and enhanced inflammatory signaling in neurons and glia [70]. Peroxisomal dysfunction can further promote neuroinflammation through impaired β-oxidation of very-long-chain fatty acids and defective plasmalogen biosynthesis, resulting in lipid accumulation, reduced antioxidant protection, and altered membrane properties that enhance microglial and astrocytic activation [5]. Reduced plasmalogen levels, altered lipid metabolism, oxidative stress, and cognitive decline have been strongly associated with Alzheimer’s disease and other neurodegenerative disorders [69,70]. Importantly, peroxisomes and mitochondria cooperate in lipid oxidation, redox regulation, and organelle quality control; therefore, dysfunction in one organelle may exacerbate stress in the other, forming an inter-organelle feed-forward loop that contributes to neuronal degeneration [69,70]. Consequently, peroxisomal redox regulation, catalase activity, plasmalogen metabolism, very-long-chain fatty acid handling, and pexophagy are emerging therapeutic targets for neurodegenerative diseases, although further translational validation is still required [69].

### 4.4. Coupling Between Aggregation, Autophagy, and Cell Death

The progressive failure of proteostasis is characteristic of NDs, resulting from the interaction between protein aggregation and impaired degradation systems. The accumulation of misfolded proteins, including amyloid-β, tau, α-synuclein, and mutant huntingtin, in neurons leads to the formation of toxic oligomers and fibrillar aggregates that disrupt cellular homeostasis. These aggregates disrupt intracellular transport, mitochondria, and synaptic signaling, increasing cellular stress [36]. One of the major cellular defense mechanisms that removes impaired proteins and organelles is autophagy. Nevertheless, under neurodegenerative conditions, autophagic mechanisms are impaired due to either autophagosome malformation, lysosomal breakdown, or impaired cargo recognition [71]. The condition causes the build-up of unprocessed substances, which puts more strain on the cellular space. More importantly, protein aggregation and autophagy dysfunction are bidirectionally linked. The protein aggregates may suppress the autophagic process and interfere with lysosomal activity, whereas defective autophagy may accelerate the accumulation of toxic proteins [72]. The result of this mutual relationship is a self-reinforcing loop that leads to gradual proteostatic breakdown and cell death. From a therapeutic perspective, the key issue is not simply whether protein aggregates are present, but whether clearance pathways remain functionally competent [73]. Increasing autophagy initiation without improving lysosomal degradation may increase autophagosome accumulation and worsen proteostatic stress [73]. Conversely, enhancing lysosomal biogenesis, cargo recognition, and mitophagy may reduce aggregate burden and improve mitochondrial quality control. Thus, aggregation–autophagy coupling represents a rational polypharmacological target because it links toxic protein clearance, organelle turnover, inflammatory signaling, and cell-death regulation within a single disease-relevant network [74].

### 4.5. Synaptic–Immune Crosstalk and Network Vulnerability

Synaptic dysfunction is among the earliest and most functionally significant consequences of neurodegenerative processes [75]. The synapses are very vulnerable to disruptions in cellular homeostasis, as they require the accurate coordination of molecular processes and are highly demanding of cellular resources [76]. Proinflammatory cytokines, including IL-1β, IL-6, and TNF-α, disrupt synaptic transmission by altering neurotransmitter release, receptor activity, and synaptic plasticity, leading to impaired long-term potentiation and cognitive deficits [77]. Mitochondrial malfunction and oxidative stress contribute to further synapse deterioration by reducing ATP availability and exacerbating local oxidative injury. All these effects impair synaptic function and reduce synaptic connections, which subsequently lead to network-level dysfunction. Notably, synaptic damage in itself may induce immune activation [78]. The loss of synapses leads to the release of damage-associated molecular patterns (DAMPs), which activate microglia and astrocytes and drive long-term neuroinflammation. This forms a two-way feedback cycle in which inflammation leads to synaptic dysfunction, and synaptic damage triggers inflammatory mechanisms, further accelerating disease progression [79].

### 4.6. Neuroimmune, Gut–Brain, and Systemic Inflammatory Crosstalk

**Figure 4:**
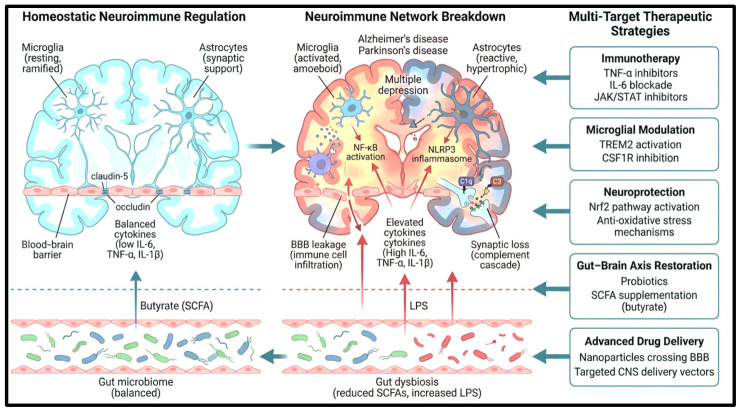
Summarizes neuroimmune circuitry under physiological and pathological conditions.

Neuroimmune crosstalk plays a central role in neurodegenerative disease (ND) progression, where chronic activation of microglia, astrocytes, and peripheral immune components promotes sustained release of pro-inflammatory cytokines, chemokines, and ROS, leading to neuronal dysfunction and degeneration [80]. Systemic factors such as gut microbiota dysbiosis and circadian rhythm disruption further modulate neuroimmune responses and intensify neuroinflammation through gut–brain axis signaling [81]. Consequently, current therapeutic strategies are shifting toward systems-based approaches targeting key regulatory pathways including NF-κB signaling, NLRP3 inflammasomes, microglial phenotypic modulation, CSF1R blockade, TREM2 regulation, and neuroprotective Nrf2 signaling to restore immune and cellular homeostasis [82]. However, disease heterogeneity, lack of reliable biomarkers, and off-target effects remain major challenges, highlighting the need for individualized and multi-target therapeutic strategies for NDs [83]. Figure 4 shows the integrated neuroimmune network in health and disease, including microglial activation, astrocytic reactivity, BBB disruption, gut–brain axis dysregulation, and emerging multi-target therapeutic interventions.

### 4.7. Shared and Disease-Enriched Mechanisms Across Major Neurodegenerative Diseases

Although commonalities such as protein aggregation, neuroinflammation, mitochondrial dysfunction, and excitotoxicity exist, each neurodegenerative disease has distinct mechanisms and vulnerable cell types, as detailed in Table 1. AChE, BACE1, tau, and Nrf2/NF-κB pathways are the major targets for polypharmacological strategies in AD, which primarily target hippocampal and cortical neurons, where amyloid-β plaques and tau tangles are primarily located [84]. PD is characterized by aggregation of α-synuclein and loss of dopaminergic neurons, which targets include MAO-B, mitophagy, and NLRP3 inflammasomes [84]. In HD, the mutant huntingtin accumulation and damage to striatal neurons have been identified as the driving forces behind the disease, and therapies must target mHTT clearance and mitochondrial support [85]. Amyotrophic lateral sclerosis (ALS) is a neurodegenerative disorder characterized by TDP-43 protein accumulation and progressive motor neuron degeneration, involving both neuroinflammatory and demyelinating mechanisms relevant to neuroimmune and polypharmacological pathways. Therapeutic strategies aim to inhibit TDP-43 aggregation, modulate glutamate signaling, and regulate the neuroimmune environment [86].

Despite the heterogeneity of symptoms, common mechanisms in NDs, such as mitochondrial dysfunction, neuroinflammation, and protein aggregation, provide a basis for polypharmacological approaches that simultaneously target multiple mechanisms. Research indicates that there are also common therapeutic targets for many NDs, as there are causal proteins for psychiatric diseases [94]. Mitochondrial dysfunction leads to oxidative stress, impaired autophagy, and neuronal damage, and mitochondrial components such as mtDNA can trigger inflammatory responses that accelerate disease progression [94]. Activated microglia, astrocytes, interleukins, and ROS further worsen neuronal loss, which further drives neuroinflammation [95]. Thus, mitochondrial quality control, protein misfolding, neuroimmune pathways, and regulators such as Mfn2 could be part of a common therapeutic approach to the treatment of several NDs [96].

### 4.8. Actionable Regulatory Hubs for Therapeutic Intervention

The pathogenic pathways involved in NDs collectively contribute to neuronal damage. NF-κB is one of the key players in inflammatory signaling, and it plays a role in chronic neuroinflammation and neuronal loss in AD and PD [97]. Neuronal injury evoked by mitochondrial stress is associated with the production of pro-inflammatory cytokines, including IL-1β and IL-18, which is believed to be regulated by the NLRP3 inflammasome, and thus it is an important target for therapeutic interventions [98]. Likewise, the cGAS–STING pathway is involved in triggering innate immune response via interferon and NF-κB signaling, which is responsible for inflammation in aging and neurodegeneration [97]. These pathways present a clear connection between mitochondrial dysfunction and neuroimmune activation in NDs.

A few other therapeutic pathways are also highlighted in Table 2, including Nrf2, the AMPK–mTOR–TFEB axis, and TREM2/CSF1R signaling. In AD and PD, Nrf2 protects against oxidative stress by inducing antioxidant and detoxifying genes, thus providing a beneficial role in modulating antioxidant defense [99]. The AMPK–mTOR–TFEB pathway is responsible for the regulation of autophagy and lysosomal activity, which is responsible for the removal of toxic protein aggregates like amyloid-β and α-synuclein; protein accumulation is involved in the pathogenesis of NDs and is linked to the dysfunction of the AMPK–mTOR–TFEB pathway [98]. TREM2 and CSF1R are important regulators of microglial activity, and they are essential to the balance between protective and pro-inflammatory microglial states, which are crucial for neuroinflammation and disease progression [100]. These actionable hubs offer a promising avenue for multi-target therapies to enter the therapeutic landscape for NDs.

## 5. Polypharmacology as a Systems-Level Therapeutic Strategy

The ability of polypharmacology to discover drugs that target multiple proteins can play a pivotal role in the development of therapeutics for complex diseases such as cancer, NDs, and metabolic disorders. The approach aims to create multi-target drugs that can influence multiple disease-relevant targets, thereby improving therapeutic efficacy compared with classic single-target drugs. One of the most significant advances in this area is the creation of MTDLs that could target multiple aspects of a disease, providing a more comprehensive therapeutic effect and reducing side effects [103]. Combination therapy and network pharmacology are also important drug modalities in polypharmacology, alongside MTDLs. Combination therapy involves using several drugs that target different disease pathways, and is becoming an increasingly popular treatment in cancer and other complex diseases [104]. Network pharmacology can help elucidate drug interactions within the complex disease gene network and enable more specific drug design [105]. Moreover, drug repurposing is a well-functioning approach towards finding new applications for existing drugs [103]. Polypharmacology is deeply interconnected with computational methods, as detailed applications are listed in Table 3, including AI and epigenetic polypharmacology, leading to a systems-level perspective on disease and treatment options [106,107]. Dual-target and multi-target agents have been designed using an AI-based drug discovery approach, which is based on AI and Computational Biology [106].

**Figure 5:**
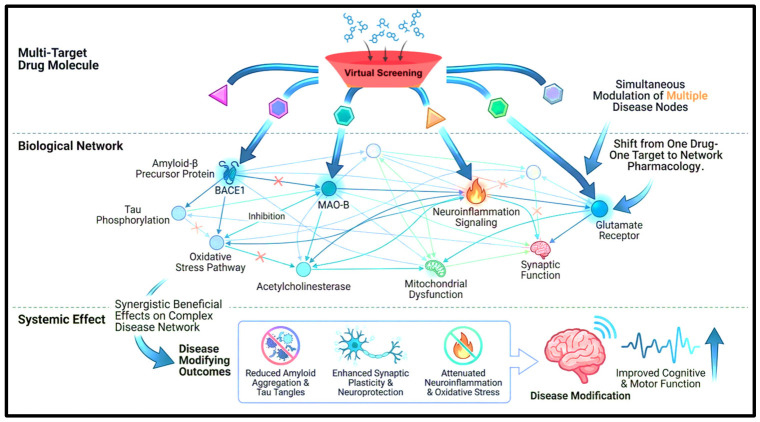
Systems-Oriented Polypharmacology in Neurodegenerative Disorders: Network-Based Therapeutic Strategies for Multi-Target Modulation and Restoration of Neuronal Homeostasis.

It has been proposed that multiple processes are involved in NDs, including oxidative stress, neuroinflammation, mitochondrial dysfunction, impaired proteostasis, excitotoxicity, and abnormal protein aggregation, all of which contribute to promoting neuronal damage and synaptic loss [24]. These pathways communicate with each other through positive feedback loops, so that single-target therapies do not effectively yield clinical responses. Thus, the concept of polypharmacology has appeared as a systems-based approach, which involves targeting multiple disease pathways simultaneously with the help of network pharmacology and systems biology [25]. The discovery of antioxidant, anti-inflammatory, and neuroprotective activities of multi-target directed ligands (MTDLs) and hybrid molecules is facilitated by the use of modern computational tools, to identify key molecular hubs associated with oxidative stress, inflammation, mitochondrial functions, and proteostasis [111]. This systems-based paradigm is shown in Figure 5, which illustrates the shift from single-target pharmacology to network-based polypharmacological intervention. It highlights (i) the interconnectedness of pathological networks of neurodegeneration, such as oxidative stress, mitochondrial dysfunction, neuroinflammation, and impaired proteostasis and protein aggregation, (ii) the positive-feedback mechanisms through which neurodegeneration spreads, and (iii) the relative therapeutic approaches whereby traditional single-target drugs only alter a small number of pathways, multi-target. The use of drug repurposing and combination therapy also plays a role in this approach; however, problems with doses and BBB permeability persist [24].

### 5.1. Limitations of Single-Target Therapy in Neurodegeneration

Single-target interventions may produce measurable biological effects, but they often fail to achieve durable disease modification in heterogeneous NDs because pathogenic signaling networks are redundant, compensatory, and stage-dependent. Inhibition of one molecular pathway may be bypassed by parallel inflammatory, metabolic, proteostatic, or synaptic mechanisms [112]. For example, reducing one upstream pathological trigger may not sufficiently suppress downstream mitochondrial dysfunction, glial activation, synaptic injury, or impaired protein clearance once these processes become self-sustaining [113]. However, this limitation should not be interpreted as evidence that all selective therapies are ineffective. Highly pathway-specific or target-specific interventions may remain valuable in genetically defined, early-stage, or biomarker-selected patient subgroups. Therefore, the goal of polypharmacology is not to abandon target selectivity, but to identify when coordinated modulation of several disease-relevant nodes is more appropriate than intervention at a single molecular target.

The endocannabinoid system is a sophisticated lipid-signaling network that helps maintain neural homeostasis by regulating synaptic transmission, immune responses, energy metabolism, and neuronal survival [114]. It comprises endogenous ligands, including anandamide and 2-arachidonoylglycerol, cannabinoid receptors CB1 and CB2, and enzymes involved in the degradation of the ligand, including FAAH and MAGL. The prevalence of this system in most neurons, glial cells, and immune cells makes it one of the most important regulatory centers capable of integrating a wide range of physiological and pathological signals. ECS dysfunction is linked to augmented neuroinflammation, excitotoxicity, and oxidative stress in neurodegenerative illnesses, which is the reason why it was chosen as a highly appealing therapeutic goal in polypharmacological intervention [115].

### 5.2. Polypharmacology and Multitarget Drug Design in the Endocannabinoid System

**Figure 6:**
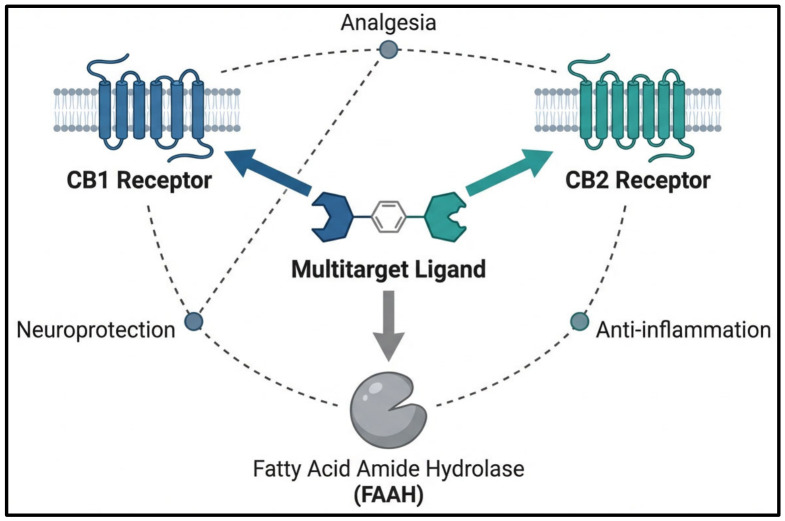
Mapping Direct Binding Interactions in Multitarget Drug Design for the Endocannabinoid System.

Polypharmacology refers to the intentional modulation of multiple disease-relevant targets or pathways by a single agent or therapeutic strategy. Multi-target-directed ligands are single chemical entities designed to engage two or more defined molecular targets [116]. Combination therapy uses two or more separate agents to regulate complementary mechanisms, but it introduces challenges related to dose optimization, pharmacokinetic compatibility, and drug–drug interactions [117]. Network pharmacology is not itself a therapy; rather, it is a discovery and analysis framework that integrates disease genes, protein–protein interactions, pathway enrichment, and drug–target relationships to identify actionable disease modules. Drug repurposing identifies new neurotherapeutic applications for approved or clinically characterized compounds based on known or newly recognized target profiles [118]. These concepts overlap but should not be used interchangeably, because each has different implications for drug design, evidence interpretation, safety evaluation, and clinical translation. Mapping Direct Binding Interactions in Multitarget Drug Design of the Endocannabinoid System, which provides an overview of the network interactions among various drug design strategies (polypharmacology, hybrid molecules, prodrugs, allosteric modulators, and biased agonism) with key endocannabinoid system components (CB1 and CB2 receptors, FAAH, MAGL) [119]. The interrelation between these targets and how multitarget strategies can simultaneously tune multiple signaling pathways is shown in Figure 6.

Phytocannabinoids like cannabidiol are an example of natural polypharmacology since they can react with various molecular targets other than the classical cannabinoid receptors [120]. CBD modulates serotonin receptors, ion channels, and inflammatory pathways, and indirectly affects the activity of ECS enzymes. This broad receptor specificity underlies its neuroprotective, anxiolytic, and anti-inflammatory activities, with minimal psychoactive effects. The aforementioned properties render CBD a promising scaffold for the development of multi-target drugs. Nevertheless, the dynamics of ECS signaling and its interactions with other biological systems are complex, making the optimization of therapeutic response and the reduction of side effects highly dependent on advanced pharmacokinetic models and systems-level analysis [121].

### 5.3. Representative Multi-Target-Directed Ligands and Molecular Hybridization Strategies

Multi-target therapies have received increasing attention in the treatment of NDs, particularly MTDLs. The goal of these strategies is to target multiple disease pathways (e.g., oxidative stress, neuroinflammation, amyloid aggregation, and mitochondrial dysfunction) simultaneously to improve therapeutic outcomes. Several different hybrid molecules and multi-target compounds are being explored for their ability to regulate multiple, interconnected processes in pathological conditions. These therapies aim to bypass the limitations of treatments that target a single factor, which are insufficient to address the multifactorial nature of these diseases. For instance, FAAH/MAGL inhibition has more potent effects on endocannabinoid tone than selective inhibition of either enzyme alone, resulting in increased anti-inflammatory and neuroprotective effects. Furthermore, multi-target strategies targeting oxidative stress control and mitochondrial stability have yielded encouraging results, particularly since these processes are important for neuronal survival. Yet, despite their therapeutic potential, difficulties with receptor desensitization and systemic pharmacodynamics, as well as their psychoactivity stemming from CB1 effects, remain. A recent study aims to develop peripherally restricted compounds and CB2-selective agents to harness therapeutic potential and mitigate side effects [122]. Representative multi-target strategies for NDS are summarized in Table 4, including evidence level, BBB considerations, and the limitations of these therapies. These include cholinesterase inhibitors, hybrid molecules, and natural-product-inspired scaffolds like curcumin, resveratrol, and quercetin, which target several pathological events associated with neurodegeneration.

### 5.4. Drug Repurposing and Combination-Based Multi-Target Therapy

**Figure 7:**
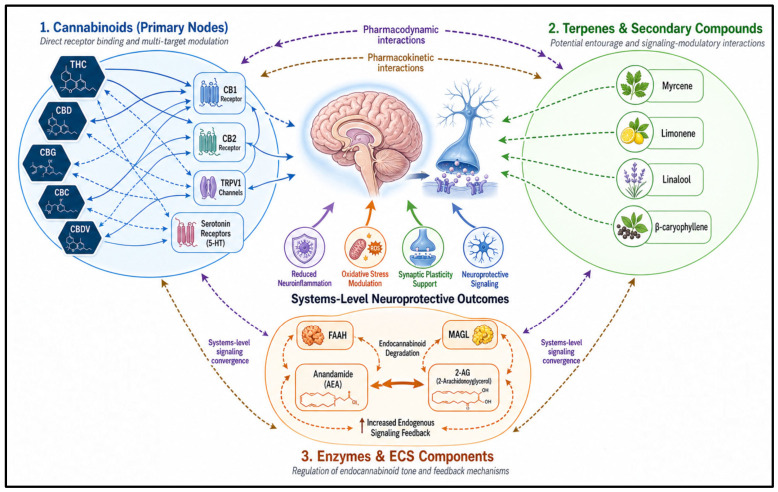
Endocannabinoid System—Related Multi-Target Modulation as a Conceptual Framework for Network Pharmacology.

The entourage effect refers to the synergistic interaction of cannabinoids and other lipid mediators that produces enhanced therapeutic effects compared to individual compounds. This synergy arises through multicomponent biochemical mechanisms, including receptor modulation, enzyme inhibition, and enhanced lipid signaling. Cannabinoid combinations such as THC and CBD show stronger neuroprotective, anti-inflammatory, and cognitive benefits in NDs like ADs [131]. The model of integrated cannabinoid synergy in the endocannabinoid system is presented as a network-based model (Figure 7) and emphasizes interactions among phyto-cannabinoids, terpenes, and endogenous signaling elements.

The network also shows how these interactions combine to produce major neuroprotective responses, including neuroinflammation, oxidative stress, synaptic plasticity, and neuronal integrity. Notably, the figure highlights the roles of direct binding interactions and indirect synergistic pathways, showing that multi-component cannabinoid preparations can act on multiple molecular targets simultaneously. This integrative model emphasizes the utility of integrated treatment methods over single-target therapies, especially in complex neurological conditions. Multicannabinoid interactions at the cellular level control neuronal excitability, glial activation, and immune responses, thus reestablishing neuronal network stability. These effects help to increase the neuroprotection, improve mitochondrial activity, and decrease protein aggregation. Moreover, when combined with receptor modulation, endocannabinoid signaling can be fine-tuned through enzyme inhibition, helping prevent overstimulation and side effects. This combined therapy approach is a promising future trend in therapeutic development, especially when paired with recent advances in technology such as AI-based drug design and nanoparticle-based CNS delivery models [132].

### 5.5. Endocannabinoid System Modulation as a Representative Polypharmacology Case Study

The endocannabinoid system (ECS) is a unique and complex regulatory system implicated in the control of various cellular processes in the CNS, making it an attractive target for polypharmacological interventions in neurodegenerative disorders. The ECS plays a key role in regulating synaptic transmission, neuroinflammation, oxidative stress, glial activation, and metabolic signaling, all of which are pivotal to the pathogenesis. The ECS’s ability to modulate neuroinflammation via the CB2 receptor, which is primarily expressed on microglial cells, is one of its most important therapeutic properties. CB2 receptor activation lowers pro-inflammatory cytokine levels, blocks NF-κB signaling, and shifts microglia from a pro-inflammatory (M1) to a neuro-protective (M2) state, thereby decreasing tissue damage and supporting tissue repair mechanisms. Also, FAAH (Fatty Acid Amide Hydrolase) and MAGL (Monoacylglycerol Lipase) inhibition promotes the release of endocannabinoids, enhances neuroprotective signaling pathways, and reduces excitotoxic glutamate release, thereby reducing oxidative stress.

There are, however, obstacles to ECS-based therapies, such as CB1-mediated psychoactivity, dose-dependent effects, receptor desensitization, and incomplete clinical validation. Nevertheless, dual FAAH/MAGL inhibition has proven to be one of the most promising approaches to enhancing cannabinoid tone, enhancing synaptic plasticity, and ultimately offering neuroprotection. Furthermore, multi-target approaches (combining ECS modulation with modulation of other targets, such as cyclooxygenase (COX) or Transient Receptor Potential Vanilloid 1 (TRPV1) channels) have emerged as a promising avenue to improve therapeutic effects in chronic neurodegenerative disorders, where multiple targets and processes interact and support one another. The dual CB1/CB2 agonists have been shown to have superior neuroprotective properties in preclinical models, particularly in multiple sclerosis and PD models [133]. The multi-target approach using ECS leads to better regulation of synaptic transmission, reduced inflammation, and greater cellular resistance, resulting in effective control of immune, neuronal, and metabolic pathways. A summary of these strategies is provided in Table 5, which lists a range of polypharmacological compounds with potential for ECS applications, along with the disease context, mechanism, level of evidence, and any current limitations.

## 6. Chemical Modulation of Interconnected Signaling Networks

**Figure 8:**
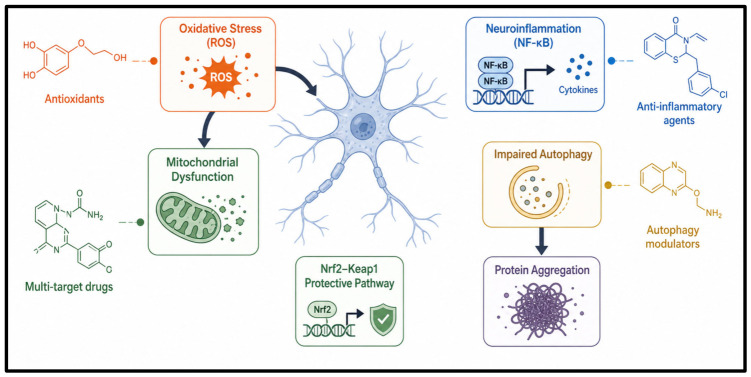
Chemical Modulation of Key Signaling Pathways in NDs.

NDs have been considered multifactorial disorders with complicated interplay between oxidative stress, neuroinflammation, mitochondrial dysfunction, impaired autophagy, and protein aggregation. The processes are strongly interdependent and constitute a dynamic network of signaling pathway crosstalk that promotes neuronal damage. The activation of inflammatory responses, including NF-κB, and the subsequent mitochondrial dysfunction caused by ROS are key factors that initiate a vicious cycle of oxidative stress and cell damage, promoting each other. Simultaneously, the Nrf2–Keap1 pathway serves as a primary regulator of the antioxidant defense mechanism, opposing oxidative damage, and its mutual connection with NF-κB signaling also regulates the equilibrium between inflammation and cytoprotection. Also, metabolic signaling is linked to autophagy control via survival pathways such as PI3K/Akt/mTOR, the dysregulation of which leads to protein retention and neuronal death. The complexity of intracellular defense mechanisms is further underscored by the functional interplay between Nrf2 signaling and autophagy, mediated by adaptor proteins like p62. Notably, these overlapping routes are a compelling argument for multi-target therapeutic approaches, since chemical modulators can act on multiple signaling cascades in parallel to reestablish cellular homeostasis and increase neuronal survival, as demonstrated in Figure 8. For instance, dual FAAH/MAGL inhibition increases endocannabinoid tone, improving synaptic plasticity and offering neuroprotective effects. CB1/CB2 dual agonists provide enhanced protection, especially in models of PD and multiple sclerosis. These multi-target approaches, as summarized in Table 6, demonstrate significant potential for treating chronic neurodegenerative conditions in which multiple pathological processes interact.

### 6.1. Redox-Inflammatory Axis Modulators

The redox-inflammatory axis is among the most actionable examples of pathway crosstalk in neurodegeneration, as oxidative stress and inflammatory signaling amplify each other through defined molecular nodes. Excessive ROS can activate NF-κB and NLRP3-dependent inflammatory responses, whereas chronic cytokine signaling further impairs mitochondrial function and increases ROS generation. In parallel, the Nrf2–Keap1–ARE pathway counterbalances this process by inducing antioxidant and cytoprotective genes. Therefore, compounds that simultaneously dampen maladaptive NF-κB/NLRP3 activity while restoring Nrf2-mediated antioxidant capacity may provide broader neuroprotection than agents targeting either oxidative stress or inflammation alone [139]. In most NDs, including AD, PD, and ALS, similar biochemical phenotypes are observed. They are oxidative stress, impaired proteostasis (autophagy and mitophagy), excitotoxicity, and chronic neuroinflammation. The integration of these mechanisms underscores the need to examine pathway integration rather than individual signaling events. The primary cause of neuronal damage is oxidative stress, frequently driven by mitochondrial dysfunction and NADPH oxidase overactivity. Phytochemicals have demonstrated beneficial neuroprotective effects, lowering ROS levels and stimulating the endogenous antioxidant defense system, such as the KEAP1/Nrf2/ARE pathway, thereby restoring redox balance and cell integrity [140].

### 6.2. Proteostasis and Autophagy Modulators

Proteostasis-targeted strategies should distinguish between inducing autophagy and fully activating autophagic flux. In neurodegenerative disease models, accumulation of autophagosomes may indicate increased initiation, impaired lysosomal degradation, or defective cargo delivery. Key regulatory nodes include AMPK–ULK1-mediated autophagy initiation, mTOR-dependent nutrient sensing, TFEB-mediated lysosomal biogenesis, p62/SQSTM1-dependent cargo recognition, and lysosomal acidification. These nodes are therapeutically relevant because impaired proteostasis also interacts with mitochondrial dysfunction and innate immune signaling; defective mitophagy can increase mitochondrial ROS and mtDNA leakage, thereby activating inflammatory pathways such as NLRP3 and cGAS–STING. Thus, effective proteostasis modulation should aim to restore flux across the full autophagy–lysosomal pathway rather than simply increasing autophagosome formation [141].

Other signaling pathways, including mTOR, Wnt, AMPK, PGC-1α, and Sirt1, are also essential to neurodegenerative mechanisms, especially in AD, in addition to cGAS-STING. These pathways control cellular metabolism, mitochondrial biogenesis, and protein synthesis, which are impaired in neurodegeneration. The synthetic or natural bioactive compounds can be used to modulate these pathways, decrease the amyloid-beta deposition, enhance the mitochondrial dynamics, and suppress neuroinflammation [142]. There are also natural compounds, including alkaloids and polyphenols, which have neuroprotective effects through regulating signaling pathways, such as JAK/STAT and IRS/PI3K. These compounds not only minimize oxidative stress but also modulate inflammatory responses, thereby enhancing neuronal survival. Moreover, the Nrf2-Keap1 axis interacts with endogenous signaling pathways, such as the hydrogen sulfide (H_2_S) pathway, thereby increasing cellular resistance to oxidative injury. The involvement of mechanotransduction pathways that convert mechanical forces into biochemical signals in ND protein aggregation is also evident. Also, non-coding RNAs (ncRNAs) have a regulatory role in the expression of genes and protein translation, and their regulation by phytochemicals is another promising therapeutic option [26].

### 6.3. Mitochondrial and Bioenergetic Modulators

NDs arise from interacting metabolic, inflammatory, proteostatic, and synaptic abnormalities; mitochondrial and bioenergetic modulators have become attractive components of multi-target therapeutic strategies. Unlike conventional drugs, which bind to a single target, the goal of MTDLs is to regulate multiple biological pathways simultaneously during disease progression. This will enable the breaking of pathological feedback mechanisms and a more holistic therapeutic action [143]. The rational drug design strategies commonly used to create MTLs combine multiple pharmacophores into a single molecular structure. These substances can address oxidative stress, neuroinflammation, mitochondrial dysfunction, and protein aggregation simultaneously. In the case of AD, MTDLs have been developed to inhibit cholinesterases, prevent amyloid-beta aggregation, control tau phosphorylation, and inhibit enzymes such as monoamine oxidase (MAO) and glycogen synthase kinase-3β (GSK-3β). Such a multi-target activity is essential due to the low effectiveness of existing treatments, which are mostly symptomatic [144]. Although they have benefits, the development of MTDL faces several challenges, including balancing efficacy across multiple targets, reducing off-target effects, and ensuring adequate bioavailability. Transportation of drugs across the BBB is a major challenge. But with the development of computational modeling, AI, and nanomedicine, the design and optimization of these compounds are becoming easier, and their potential for clinical use is increasing [145].

#### 6.3.1. Role of Natural Products in Multi-Pathway Modulation

Natural products have attracted considerable interest as multi-target therapeutic agents due to their diverse chemical structures and biological functions. Phytochemicals, including polyphenols, flavonoids, alkaloids, and terpenoids, have diverse neuroprotective properties, including antioxidant, anti-inflammatory, anti-apoptotic, and anti-aggregation effects. They are especially good at modulating the complex pathway crosstalk implicated in NDs, owing to these properties [146]. Established examples of these compounds include curcumin, resveratrol, and quercetin, which have shown the capacity to target major signaling pathways, such as NF-κB, NLRP3 inflammasome, PI3K/Akt, and Nrf2/HO-1. These mechanisms help them to alleviate oxidative stress, inhibit neuroinflammation, and improve mitochondrial activity. Also, natural products can regulate microglial activation, thereby inhibiting neurotoxicity and enhancing neuronal survival [147]. Natural products have some limitations, even though they can have therapeutic potential; they have low bioavailability, are metabolized too quickly, and are not standardized. Nanotechnology-based delivery systems are being developed to address these problems by enhancing stability, improving targeting efficiency, and improving brain penetration. There has been promising evidence from clinical studies, but additional research is necessary to optimize dosage, establish safety, and demonstrate long-term effectiveness [148].

#### 6.3.2. Synthetic Compounds and Rational Drug Design

Synthetic compounds offer a highly regulated system of targeting various pathways of neurodegeneration. Recent breakthroughs in medicinal chemistry and computational drug design have enabled the creation of hybrid molecules that combine multiple pharmacological properties into a single compound. They are compounds that are engineered to bind with more than one molecular target, thus increasing therapeutic efficacy [149]. Synthetic multi-target compounds can also serve as inhibitors of acetylcholinesterase (AChE), beta-secretase (BACE-1), monoamine oxidase (MAO), and histone deacetylases (HDACs). These are linked to the most important pathological characteristics, including amyloid-beta and tau hyperphosphorylation, neuroinflammation, and synaptic dysfunction. Such compounds are normally designed by molecular hybridization methods to enhance their pharmacokinetic characteristics and BBB penetration [150]. Nevertheless, there are still problems with toxicity, off-target effects, and low bioavailability. To address such problems, nanotechnology-derived drug delivery systems are being investigated to improve targeting specificity and minimize side effects. In general, synthetic compounds are also a promising approach for developing disease-modifying therapies.

#### 6.3.3. Integrated Chemical Strategies for Pathway Modulation

**Figure 9:**
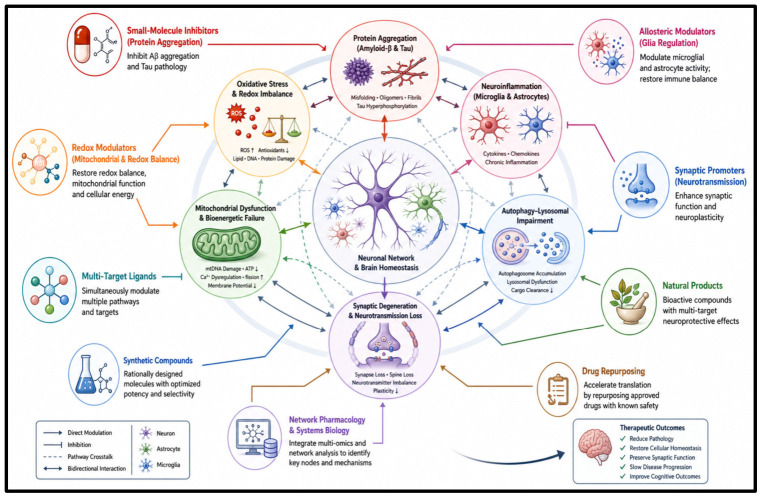
Integrated Chemical Strategies for Multi-Pathway Modulation in NDs.

The problem of modulating pathway crosstalk in NDs requires integrated chemical strategies that combine multiple therapeutic modalities, as shown in Figure 9. Small-molecule inhibitors inhibit the misfolding and aggregation of pathogenic proteins, including amyloid-β and Tau, and, as a result, neurotoxicity. Allosteric modulators are employed to control microglial and astrocytic activation and reduce inflammatory processes, thereby contributing to the amelioration of neuronal damage. Redox modulators will re-establish the cellular redox homeostasis and mitochondrial activity, counteract oxidative stress, and enhance neuronal survival. Synaptic promoters enhance neurotransmission and connectivity, helping counteract cognitive impairment associated with synaptic deterioration. These are multi-target ligands, natural products, synthetic compounds, network pharmacology, and drug repurposing. All these strategies simultaneously regulate multiple signaling pathways, thereby enhancing therapeutic outcomes.

Multi-target compounds have broad-spectrum neuroprotective effects by targeting oxidative stress, inflammation, and protein aggregation. Natural products provide low-toxicity substitutes with a variety of bioactivities, whereas synthetic compounds enable targeted, specific interventions. Network pharmacology augments such approaches by identifying optimal targets and predicting synergies. The idea of drug repurposing also accelerates therapeutic development by leveraging previously developed drugs with established safety profiles. Together, these strategies can be viewed as a transition to the polypharmacology paradigm rather than the old paradigm of one drug and one target, which is more aptly applied to NDs, which are complex and multifactorial. Further research and technological developments are likely to advance these strategies further and enhance their clinical implementation [151].

### 6.4. Network Pharmacology Approaches in Neurodegenerative Disease Research and Drug Discovery

Network pharmacology strengthens polypharmacological drug discovery by shifting the focus from isolated target lists to interconnected disease modules, integrating disease-associated genes, protein–protein interaction networks, pathway enrichment data, and drug–target interactions to identify key hub and bridge nodes as well as pathway clusters [152]. This systems-based approach is particularly valuable for complex neurodegenerative disorders, where it helps prioritize targets linking mitochondrial dysfunction, inflammation, protein aggregation, and synaptic failure, enabling the design of multi-target therapies for conditions such as Alzheimer’s and Parkinson’s disease. By combining large-scale genomics, proteomics, metabolomics, and bioinformatics data, network pharmacology provides a holistic view of molecular interactions and regulatory mechanisms [153]. However, its computational predictions must be interpreted with caution due to limitations such as incomplete databases, cell-type specificity, disease-stage variability, and the need for rigorous experimental validation [154].

Earlier studies, as mentioned in Table 7, have shown that network pharmacology can effectively identify key targets, including AKT1, CASP3, TNF, and STAT3, that are central to neuronal survival, apoptosis, and neuroinflammation. These targets are part of key signaling pathways, such as PI3K-AKT, MAPK, and TNFα, that are highly implicated in the pathogenesis of NDs. Taken together, the network of these pathways emphasizes the interconnectedness of disease mechanisms and justifies multi-target drug design over single-target interventions. Network pharmacology can be used to identify bioactive compounds, especially natural products, which have multi-pathway synergistic therapeutic effects. Moreover, network pharmacology provides an effective platform for drug repurposing, enabling scientists to discover novel therapeutic uses of existing drugs based on their network interaction patterns. Other computational methods, including molecular docking, protein–protein interaction (PPI) network analysis, pathway enrichment analysis, and molecular dynamics simulations, are also used to predict drug-target interactions and therapeutic efficacy further. These technologies allow a better insight into the drug-induced changes in complicated biological processes and enhance the effectiveness of drug discovery pipelines [155].

Although network pharmacology offers significant benefits, it faces several obstacles, including the complexity and heterogeneity of biological data, inaccuracies in databases, and the need to validate computational predictions. Nonetheless, recent developments in AI and ML are addressing these shortcomings by enhancing data integration, improving target prediction accuracy, and refining model architecture. The integration of AI-based solutions is increasing the predictive power of network pharmacology and accelerating the identification of new treatment plans. On the whole, network pharmacology presents an integrative and all-encompassing approach to the study of disease mechanisms on the systems level and has enormous potential in the creation of holistic, multi-target, and personalized therapies to NDs [156].

**Table 7:** Network Pharmacology-Based Studies and Their Contributions to Neurodegenerative Disease Mechanisms and Therapeutics.

Disease Focus	Data Sources/Tools	Key Targets/Genes	Key Pathways	Mechanistic Insights	Drug/ Compound Insights	Contribution to ND Research	Citations
General NDs	Conceptual	Multi-target networks	Systems biology	Introduced the polypharmacology paradigm	Multi-target drugs	Foundation for ND therapeutics	[157]
AD, PD	Network modeling	Genome-wide targets	Cellular interaction networks	Drug action occurs at the network level	Systems drugs	Enabled holistic ND modeling	[158]
Complex NDs	Omics integration	Multi-scale biomarkers	Systems pharmacology pathways	Integrates genomics + proteomics	Personalized drugs	Biomarker discovery in NDs	[159]
AD	TCMSP	AKT1, IL6, TNF	PI3K-AKT, MAPK	Anti-inflammatory + anti-apoptotic	Herbal compounds	Multi-target therapy validation	[160]
AD, PD	Herbal databases	Neuroactive compounds	Neurotransmitter pathways	Multi-component synergy	TCM drugs	ND multi-target drug design	[161]
NDs	Network mapping	Protein hubs	Disease interaction networks	Identifies druggable proteins	Natural products	Expands ND therapeutic targets	[162]
Brain disorders	Network modeling	Multi-protein targets	Signal transduction	Multi-target therapy	Natural compounds	ND drug discovery insights	[163]
AD	Synergistic networks	Multi-target nodes	Multi-pathway synergy	Synergistic drug effects	Herbal medicine	Improved ND therapeutic outcomes	[164]
Neurological systems	Systems pharmacology	Drug interaction nodes	Cellular signaling	Rational drug design	Multi-target drugs	Enhances ND drug efficiency	[165]
NDs	NP tools	Target clusters	Disease pathways	Combinatorial therapy	Drug combinations	Treats complex ND mechanisms	[166]
AD	NP databases	Gene targets	KEGG pathways	Database-driven predictions	Drug screening	Target identification in NDs	[167]
AD, PD	GO, KEGG, STRING	AKT1, CASP3, TNF	PI3K-AKT, apoptosis	Reduces neuronal death	Resveratrol	Multi-pathway neuroprotection	[168]
NDs	Integrated NP	Multi-target genes	Neurodegeneration pathways	Systems-level regulation	Herbal drugs	Improves therapeutic strategies	[169]
Chronic NDs	Network pharmacology	Disease genes	Causal pathways	Targets root causes	Disease-modifying drugs	Shift from symptom to cause	[170]
AD, PD	Multi-modal NP	Core genes	Multi-pathway integration	Combines experimental + computational	TCM compounds	Strong ND applicability	[10]
NDs	Medicinal plant NP	Bioactive compounds	Anti-inflammatory pathways	Identifies neuroprotective agents	Phytochemicals	Natural ND therapies	[171]
AD	Integrated NP	Multi-target genes	Omics pathways	Systems integration	Drug combinations	Precision ND treatment	[172]
AD	TCMSP, GeneCards	MAPK, APP, BACE1	Synaptic signaling	Improves cognition pathways	Donepezil	Multi-target AD therapy	[10]
NDs	NP models	Multi-target networks	Pharmacological pathways	Explains TCM mechanisms	Herbal formulas	Mechanistic ND insights	[105]
NDs	AI + NP	Target networks	Precision pathways	AI predicts interactions	Smart drug design	Improves ND drug discovery	[173]
NDs	Machine learning	Drug-target links	Interaction networks	Predicts hidden targets	AI-based drugs	Enhances ND prediction accuracy	[174]
AD	Bibliometric tools	Research hotspots	AD-related pathways	Identifies trends	Research mapping	Guides ND future research	[175]
NDs	Integrated NP	Multi-target nodes	Systems pathways	Multi-target drug action	Drug discovery	Improves ND therapeutics	[154]
NDs	Omics + NP	Disease genes	Multi-pathway	Accelerates discovery	New drugs	Faster ND drug development	[176]
Parkinson’s	Computational NP	Dopamine-related genes	Neuroinflammation	Identifies biomarkers	PD drugs	Early PD diagnosis	[177]
NDs	Bioinformatics NP	Neuroprotective targets	Survival pathways	Predicts neuroprotection	Small molecules	Drug screening	[178]
Herbal NDs	NP validation	Bioactive targets	Anti-inflammatory	Mechanism validation	Herbal medicine	Improves reliability	[179]
Aging + NDs	Multi-omics NP	Oxidative stress genes	Mitochondrial dysfunction	Aging-driven ND mechanisms	Anti-aging drugs	Precision ND therapy	[180]
NDs	Integrative NP	Drug-target networks	Precision pathways	Personalized therapy	Smart drugs	Precision medicine	[164]
NDs	Network medicine	Biomarkers	Disease networks	Identifies new targets	Drug repurposing	ND biomarker discovery	[10]
NDs	Multi-omics NP	Integrated targets	Omics pathways	Data integration challenges	Advanced drugs	Improves ND modeling	[181]

## 7. Network Pharmacology and AI-Enabled Drug Discovery

### 7.1. AI-Guided Multi-Target Ligand Discovery

**Figure 10:**
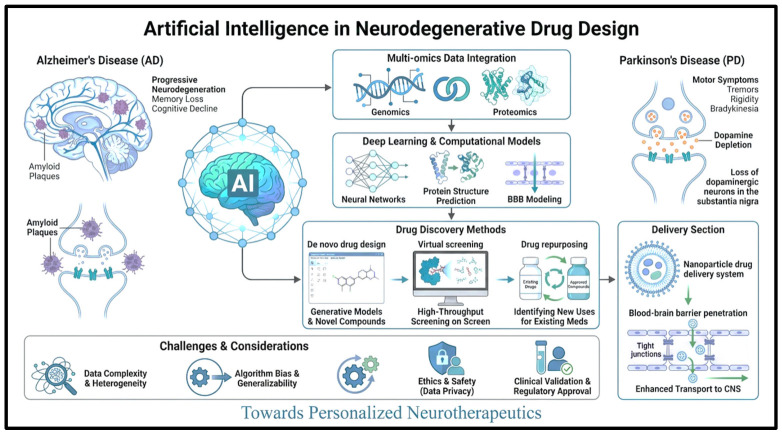
Network Pharmacology and AI-guided multi-target ligand discovery.

AI-guided drug design is transforming the field by enabling the analysis of large volumes of biological data to discover new targets and design multi-target compounds, particularly for complex diseases such as neurodegeneration, as shown in Figure 10. Machine learning and deep learning are examples of AI-based techniques that combine genomic, proteomic, and pharmacological data to identify key nodes of disease networks and forecast the molecular interactions to enable multi-pathway modulation [172]. Molecular docking, virtual screening, and QSAR modeling are tools that optimize the efficacy, selectivity, and pharmacokinetics of compounds, thereby accelerating the development of MTDLs. Drug repurposing is also improved by AI, which can identify new applications for existing drugs based on their multi-target profiles, substantially decreasing the time and cost of drug development. Also, AI developments aid the de novo design of drugs by using generative models to generate biologically active molecules with desired properties and incorporate synthesis planning [182]. Despite the data quality, model interpretability, and regulatory challenges, AI implementation in drug discovery processes holds the promise of effective, precise, and personalized medicine strategies in pharmaceutical development [183].

### 7.2. Multi-Omics, Single-Cell, and Spatial Approaches

Omics technologies, such as genomics, transcriptomics, proteomics, and metabolomics, offer a high-throughput perspective of the molecular pathways involved in the pathogenesis of NDs through the identification of complex interactions between pathways (oxidative stress, inflammation, and mitochondrial dysfunction). These technologies provide a systems-level perspective on disease progression and enable the identification of essential molecular signatures. Multi-omics data integration is beneficial for personalized medicine, as it enables patient stratification and treatment design based on their needs [184]. Further improvements in bioinformatics and machine learning enhance the capacity to integrate different layers of omics data, reveal the main regulatory nodes, and improve understanding of pathway crosstalk. Omics technologies have the potential to revolutionize systems pharmacology and hasten biomarker discovery in NDs, despite data complexity, technical constraints, and standardization problems [185].

Single-cell omics technologies enable profiling of multiple molecular layers, such as the genome, transcriptome, epigenome, proteome, and metabolome, in single cells, thereby enabling the study of cellular heterogeneity and complex biological processes. These methods can reveal interactions among molecular changes across various biological layers and provide the most important information about disease progression, development, and treatment resistance. It has been applied in the study of cell lineage, tissue atlases, tumor immunology, and mapping the spatial cellular environment [186]. In addition to these developments, microfluidic technologies complement data acquisition from single cells in a controlled microenvironment by enabling precise isolation, manipulation, and high-throughput processing. Other platforms, including droplet-based and microwell systems, are more sensitive, less prone to contamination, and capable of analyzing multiple omics simultaneously. Additional integration with sequencing and mass spectrometry technologies makes the profiling comprehensive and enhances reproducibility, whereas spatial multi-omics methods retain the tissue context [187]. Although they are associated with technical and cost-related difficulties, these technologies are already changing the face of biomedical research by enabling high-resolution molecular insights.

### 7.3. Nanomedicine and BBB-Oriented Delivery Strategies

Nanomedicine offers novel approaches that can be used to overcome the BBB, which is a significant constraint to treating, by delivering drugs to the brain using nanoparticles, including liposomes, dendrimers, polymeric carriers, and inorganic nanomaterials [150]. Such nanocarriers are capable of carrying therapeutic agents and employing the mechanisms of receptor-mediated transcytosis and ligand-based targeting to enhance the bioavailability and targeting of the drugs [188]. Also, nanoparticles enable controlled, sustained drug delivery, minimize systemic toxicity, and facilitate multi-target therapeutic approaches to oxidative stress, inflammation, and protein aggregation. The most advanced delivery methods include intranasal delivery, biomimetic nanocarriers, and ligand-functionalized nanoparticles designed to target specific brain cells. Focused ultrasound, magnetic targeting, and chemical modification methods also have a beneficial effect on BBB penetration and distribution of drugs [189]. Although there are certain difficulties connected with toxicity, scalability, and clinical translation, nanomedicine has great potential to enhance clinical outcomes and assist in detecting diseases in their early stages because of nanoscale biosensors [190].

## 8. Challenges and Limitations in Translational Polypharmacology

The profound scientific and translational challenges of NDs, particularly those related to pathway crosstalk, stem from the highly dynamic, interconnected nature of the underlying molecular processes. Interactions among critical pathways, such as autophagy, oxidative stress, endoplasmic reticulum (ER) stress, and neuroimmune signaling, not only drive disease progression but also complicate therapeutic interventions, since the regulation of one pathway can cause undesirable effects in other pathways [139]. Non-coding RNAs, including miRNAs and lncRNAs, are also regulatory molecules that can affect multiple gene networks simultaneously and are characterized by significant challenges in delivery, specificity, and off-target effects [191]. Moreover, protein aggregates such as amyloid-β, tau, and α-synuclein are synergistic and require multi-target therapy, yet developing such interventions is challenging without causing side effects. Additional mechanistic uncertainty is introduced by disruptions in organellar crosstalk among mitochondria, lysosomes, the ER, and peroxisomes, especially in a few cases of neurodegenerative disorders in which pathophysiological mechanisms remain incompletely understood. In addition, barriers such as limited BBB permeability and inter-individual variability can significantly impede the efficacy of therapeutic measures designed to regulate pathway crosstalk [192].

### 8.1. Biological Heterogeneity and Patient Stratification

The complexity and close interconnectedness of NDs are exemplified by the multi-pathway involving oxidative stress, inflammation, mitochondrial dysfunction, protein aggregation, and synaptic failure, which are interdependent through dynamic feedback loops. Such a high level of interdependence usually makes the single-pathway approaches to therapy ineffective or even counterproductive. This is further complicated by the heterogeneity of diseases and individual patients, due to genetic predisposition, environmental exposures, and lifestyle factors, making the development of a universally effective treatment difficult. Systems biology approaches have identified overlapping molecular networks of defective proteostasis, neuroinflammation, and impaired DNA repair, all of which contribute to disease progression. In addition, inter-organ communication via axes, including the gut–brain axis and immune-metabolic interactions, contributes to disease effects, with consequences for systemic inflammation and dysfunctional neurons [193]. Neuronal survival and degeneration also depend strictly on interactions among programmed cell death mechanisms, such as apoptosis, autophagy, and necrosis. Taken together, they indicate the need to consider integrative, individual, and multi-target treatment approaches that account for biological complexity and individual variability [194]. Recent translational evidence in Parkinson’s disease further illustrates that therapeutic outcomes are shaped not only by neural circuit dysfunction but also by systemic and metabolic risk profiles. For example, Yao, et al. [195] that metabolic risk factors influence deep brain stimulation efficacy in PD-related sleep and depressive disorders, supporting the need to integrate clinical phenotype, metabolic status, and individualized patient stratification when evaluating advanced neurotherapeutic interventions.

### 8.2. Off-Target Toxicity and Safety

Although multi-target therapeutic strategies are needed to treat the multifactorial nature of NDs, they are inseparably linked with a higher risk of off-target toxicity. These unintended interactions with non-disease-related biological targets may disrupt normal neuronal functions and compromise therapeutic safety [196]. This is especially important in the central nervous system, where molecular control is strictly required to sustain normal physiological functions, and long-term alteration of immune or inflammatory responses can cause immunosuppression or chronic inflammation [197]. To reach the best balance between the therapeutic effect and safety, it is important to consider drug specificity, dosage, and pharmacokinetic properties. Even though multi-target drugs can offer benefits over single-target therapies by targeting multiple disease mechanisms simultaneously, their design should account for potential compensatory biological responses and off-target interactions. Computer-aided methods such as molecular docking and pharmacokinetic modeling are currently used to predict and prevent off-target effects during drug development. However, the reduction of toxicity and the maintenance of therapeutic effects are an important issue, which also demands interdisciplinary assessment methods that are rigorous [198].

### 8.3. BBB and Delivery Barriers

One of the most significant barriers to the treatment of NDs is the BBB, which severely limits the number of therapeutic agents delivered to the central nervous system and thus limits the efficacy of drugs. The BBB is a dynamic structure in which permeability may vary throughout the disease progression, and in some cases, enhances neurodegeneration due to augmented inflammation and vascular dysfunction [199]. To overcome them, several innovative drug delivery technologies have been created, such as ligand-functionalized nanoparticles, bispecific antibody-carrying systems, focused ultrasound-mediated BBB disruption, intranasal delivery routes, and gene-editing vectors [190]. The most promising aspect of nanoparticle-based delivery systems is their ability to improve drug stability, target specific brain regions, and release drugs in a controlled manner, but the challenges of immunogenicity, toxicity, and large-scale production remain obstacles to clinical adoption. New approaches like transient BBB opening, carrier-mediated transport, and intranasal delivery can improve drug delivery to the brain. These methods enhance both safety and efficiency. Overcoming BBB limitations requires continued innovation. Interdisciplinary collaboration is essential for effective drug delivery.

### 8.4. Translational Limitations and Biomarker Gaps

Although there has been great progress in understanding the molecular etiology of NDs and in establishing novel multi-target strategies, clinical translation has remained a significant obstacle. A large number of therapeutic candidates that show efficacy in preclinical models do not succeed in clinical trials because they do not translate from animal models to human disease biology, due to patient heterogeneity and the lack of reliable early diagnostic biomarkers [200]. The complexity of NDs is also an extrinsic factor that complicates the design of clinical trials, setting of appropriate dosing schedules, treatment length, and the most effective combinations of therapeutic agents and regulatory considerations, and the high cost of development further limits such trials [201]. Additionally, the issue of delivery, including BBB, continues to hamper the effectiveness of the therapeutic process, although new technologies, including nanoparticle-based systems and focused ultrasound, promise to change the status quo. Precision medicine strategies that include patient selection guided by biomarkers and technologies to deliver drugs to patients are also increasingly being viewed as key to achieving better clinical outcomes. To bridge the gap between laboratory research and clinical application, we will need stronger translational models, improved study design, greater interdisciplinary collaboration, and a shift toward personalized therapeutic approaches.

The polypharmacological approaches have been very promising in preclinical studies, but there are many hurdles in the translation of such strategies to clinical treatments of NDs [202]. Therapies targeting antioxidants and mitochondrial function have shown early potential to mitigate oxidative stress and improve mitochondrial function, but have struggled with dosages, BBB penetration, and patient variability [203]. The natural compounds of particular interest are curcumin and resveratrol, which have multiple pathways associated with oxidative stress and inflammation; both compounds have been shown to have inconsistent bioavailability and efficacy to date [204]. Moreover, the usage of ECS-targeted strategies has been limited by the problems of psychoactivity, receptor desensitization, and difficulties in disease-specific dosing [205]. These examples are key to enhancing clinical outcomes and are critical when combined with patient selection using biomarkers and the use of multi-target therapies. Singh, et al. [206], discuss new developments in drug delivery systems, such as nanoparticles and hybrid molecules, that could enhance the delivery of drugs into the CNS. Despite this, there is still much to do to overcome translational challenges and make multi-target drugs effective in treating the complex pathophysiological processes underlying NDs.

## 9. Future Perspectives

The therapeutic approaches to NDs in the future are shifting toward systems-level therapies that target interconnected pathological networks, including oxidative stress, inflammation, mitochondrial dysfunction, and protein aggregation, by regulating key regulatory hubs rather than individual molecules. Systems biology, network pharmacology, and quantitative systems pharmacology models enable the identification of such crucial nodes and facilitate the development of multi-target interventions that help restore cellular homeostasis more efficiently. At the same time, precision medicine combines genetic, molecular, and clinical information to stratify patients based on distinct disease profiles and offer customized treatment to meet the heterogeneity in genetics, environment, and disease stage [207]. Such an individualized approach is fundamental because NDs and patients are heterogeneous, enhancing the effectiveness of therapy by aligning interventions with specific biological processes. New models also highlight the importance of multiple-organ interactions and dysfunctions in the system in the process of the development of the disease, and future treatments might require consideration of systemic factors other than those innate to the brain [208]. Collectively, systems-level knowledge and precision medicine would transform treatment paradigms, enabling early detection of disorders, targeted treatment using biomarkers, and multi-target therapy tailored to the patient in neurodegeneration.

The introduction of new technologies, including AI, multi-omics systems, and nanomedicine, will revolutionize ND management by helping detect disease early, accurately stratify patients, and provide specific treatment. AI can accelerate drug discovery by rapidly identifying multi-target compounds, predicting drug-target interactions, and optimizing pharmacological profiles; in addition, AI can enhance biomarker sensitivity and improve predictive modeling of diseases. Multi-omics methods combine genomic, transcriptomic, proteomic, and metabolomic data to identify new biomarkers and treatment targets, enhance early diagnosis, and enable individualized treatment [10]. Nanomedicine can address challenges such as the blood–brain barrier, enabling the delivery of drugs to specific parts of the brain or to specific cell types, and AI-controlled nanoparticle design can be even more effective and safer. The interplay between these technologies is conducive to the development of combination therapies and polypharmacological agents that simultaneously modulate multiple disease pathways. Nevertheless, to achieve successful clinical translation, it is necessary to enhance the quality of experimental models, sound trial designs, and multidisciplinary collaboration, and to focus on aspects such as biocompatibility, algorithmic bias, data heterogeneity, and regulatory clarity. The combination of multi-target approaches, modern technologies, and personalized medicine is promising for creating effective disease-modifying therapies that can slow disease progression and repair neuronal function in NDs [209].

## 10. Limitations

This article is a narrative review, and selection bias in the literature is possible with this type of review. The subjectivity of a narrative review means that the included studies may not capture all relevant perspectives, although we have tried to include the most recent mechanistic, translational, and computational studies. Furthermore, the review is not a systematic meta-analysis of the therapeutic effects of polypharmacological approaches in NDs. By design, a meta-analysis provides a more inclusive and quantitative summary of the available clinical and preclinical evidence, and other important studies may have been missed that would add context and/or a more complete picture.

In addition, the diseases reviewed in this review paper, AD, PD, HD, and ALS, have unique and complex pathophysiologies. They are highly variable diseases in terms of their initiating lesions, susceptible cell types, disease progression rates, and marker identification. Thus, the common network mechanisms observed across these diseases do not necessarily imply that the same therapeutic approaches will be successful for all neurodegenerative diseases. The mechanisms of action for each disease differ, and the overlap observed in this review should be interpreted with caution, as therapeutic approaches must be specific to the disease to account for these differences.

Furthermore, many of the polypharmacological strategies reviewed are based on preclinical studies, computer simulations, and early translational research. These strategies have great potential but are not yet proven or widely used in the clinic. Delivery across the BBB, safety profiles, pharmacodynamic monitoring, and the natural heterogeneity of patient populations remain big challenges. The blood–brain barrier, for instance, poses a challenge to the effective delivery of many promising therapies, thereby reducing the bioavailability of drug candidates within the CNS. Additionally, the safety of multi-target therapies is unclear, as combining multiple mechanisms may lead to unknown side effects or toxicities not seen with single-target therapies. Monitoring pharmacodynamics is also difficult in clinical trials, especially in long-term trials with complex polypharmacological interventions. Another challenge to clinical trial design and interpretation is patient heterogeneity, particularly regarding genetic profiles, comorbidities, and disease stages of NDs. Further, the design of trials, such as the selection of appropriate biomarkers, dosing regimens, and outcome measures, is a key factor in the successful clinical translation of these therapeutic strategies.

The combination of polypharmacological strategies presented in this review represents a new and exciting area of potential therapeutic development for NDs, but it is still in its infancy. The effectiveness of these approaches will depend on overcoming many challenges, including delivery barriers, safety concerns, the use of biomarkers to demonstrate effectiveness, and ensuring they meet the needs of diverse patient populations. The findings reported in this review need to be considered in the context of a larger body of evidence; however, further clinical testing and systematic studies are needed to determine the efficacy of these multi-target therapeutic applications in clinical practice.

## 11. Conclusions

Neurodegenerative diseases (NDs) are now recognized as complex network disorders involving interconnected pathways such as oxidative stress, neuroinflammation, and protein aggregation. In addition to mitochondrial dysfunction, peroxisomal abnormalities should be recognized as part of the broader organelle-stress network that contributes to oxidative damage, lipid disruption, neuroinflammation, and disease progression. This complexity limits the effectiveness of single-target therapies, although selective treatments may still benefit specific patient subgroups. In contrast, polypharmacology presents a more biologically coherent and therapeutically relevant foundation for treatment. By enabling the concurrent modulation of multiple disease-related nodes, pathways, and regulatory centers, polypharmacology more closely aligns with the complexity of diseases themselves. Approaches such as multi-target-directed ligands, molecular hybridization, rational combination therapy, natural-product-inspired scaffolds, and drug repurposing hold substantial promise in restoring cellular homeostasis. Rather than merely inhibiting isolated pathological processes, these approaches offer the potential to re-establish balance across several interconnected biological systems. Such comprehensive strategies are crucial for tackling the diverse and multifaceted nature of NDs. Simultaneously, the future of neurodegenerative drug discovery is undergoing a significant transformation, driven by advancements in network pharmacology, AI, multi-omics profiling, single-cell technologies, and nanomedicine. These cutting-edge tools are poised to revolutionize the way we approach disease mechanisms. They facilitate the identification of convergent molecular targets, support the design of multifunctional compounds, and enable biomarker-guided patient stratification, all of which are essential for addressing major clinical challenges, including those related to BBB penetration. Despite these advancements, significant hurdles remain, including disease heterogeneity, translational fidelity gaps in preclinical models, safety concerns with multi-target interventions, the absence of reliable clinical biomarkers, and the regulatory complexity of developing novel therapies. In review, future neurodegenerative disease treatment will rely on multidisciplinary and patient-specific approaches. A shift toward network-based polypharmacology may enable safer, more effective, and personalized therapies for NDs.

## Figures and Tables

**Table 1:** Disease-specific mechanisms and potential multi-target focus in major NDs.

Disease	Main Protein/ Pathology	Vulnerable Cells	Enriched Mechanisms	Possible Multi-Target Focus	References
Alzheimer’s Disease (AD)	Aβ, tau	Hippocampal/cortical neurons	Synaptic failure, inflammation, cholinergic loss	AChE/BACE1/tau/Nrf2/NF-κB, Sirtuin activation, Neuroinflammation	[87]
Parkinson’s Disease (PD)	α-synuclein	Dopaminergic neurons	Mitochondrial dysfunction, lysosomal failure	MAO-B/α-synuclein/mitophagy/NLRP3, PINK1/PARKIN signaling, α-synuclein fibrilization inhibition	[88]
Huntington’s Disease (HD)	Mutant huntingtin (mHTT)	Striatal neurons	Transcriptional dysregulation, excitotoxicity	mHTT clearance, mitochondrial support, HDAC inhibitors, AMPA receptor antagonism	[89]
Amyotrophic Lateral Sclerosis (ALS)	TDP-43, SOD1/C9orf72	Motor neurons	RNA defects, axonal transport, and inflammation	TDP-43, glutamate, neuroimmune modulation, autophagy enhancers	[90]
Frontotemporal Dementia (FTD)	TDP-43, FUS, tau	Cortical neurons	RNA-binding protein dysfunction, neuroinflammation	TDP-43 clearance, tau phosphorylation, and neuroinflammation inhibition	[91]
Lewy Body Dementia (LBD)	α-synuclein, tau	Dopaminergic neurons, cortical neurons	α-synuclein aggregation, neuroinflammation	MAO-B inhibition, α-synuclein aggregation inhibitors, NLRP3 modulation	[92]
Multiple Sclerosis (MS)	Oligodendrocyte damage, demyelination	Oligodendrocytes, motor neurons	Autoimmunity, neuroinflammation, and BBB dysfunction	Immunomodulators (glatiramer acetate), neuroprotection, myelin repair	[93]

**Table 2:** Actionable Regulatory Hubs and Potential Targets for Therapeutic Intervention.

Regulatory Hub	Disease Mechanism	Targeted Pathways	Potential Therapeutic Intervention	Citations
NF-κB	Inflammatory signaling in NDs	Inflammation, neuronal loss, and synaptic dysfunction	NF-κB inhibitors to reduce neuroinflammation	[97]
NLRP3 Inflammasome	Mitochondrial dysfunction, neuroinflammation	Cytokine maturation, activation of IL-1β, IL-18	NLRP3 inhibitors to suppress inflammasome activation	[98]
cGAS–STING	Mitochondrial damage, neuroinflammation, and DNA sensing	Cytosolic DNA, mitochondrial DNA leakage	cGAS–STING inhibitors to reduce inflammation and neurodegeneration	[101]
Nrf2	Antioxidant defense, oxidative stress regulation	Redox balance, cellular protection	Nrf2 activators to promote neuroprotection	[99]
AMPK–mTOR–TFEB Axis	Autophagy, lysosomal control, proteostasis	Autophagy activation, lysosomal function	AMPK activators, mTOR inhibitors, and TFEB modulators to enhance autophagy	[102]
TREM2/CSF1R Signaling	Microglial activation, neuroinflammation	Microglial phenotype modulation	TREM2/CSF1R agonists to promote neuroprotective microglia	[102]

**Table 3:** Key Terms and Their Applications in Polypharmacology.

Term	Explanations	Examples	Description	Citations
Polypharmacology	Multi-target modulation strategy	Broad therapeutic principle	A strategy targeting multiple disease pathways with a single drug.	[103]
MTDLs	One molecule, multiple targets	AChE/MAO-B/BACE1 inhibitors	Compounds designed to target multiple molecular pathways.	[108]
Combination Therapy	Multiple drugs are used together	Antioxidant + anti-inflammatory therapy	Using multiple drugs in tandem to target different disease mechanisms.	[103]
Network Pharmacology	Computational mapping of disease/drug networks	Target prioritization	A systems approach to understanding drug-target interactions across multiple biological pathways.	[104]
Drug Repurposing	New use for an existing, approved drug	Existing drugs tested for neuroprotection	Identifying new therapeutic uses for already approved drugs.	[103]
Multi-target drugs	Drugs designed to interact with more than one biological target	Cancer treatment agents	Drugs that simultaneously target multiple biological pathways to enhance efficacy.	[104]
Polytherapy	Use of multiple drugs targeting different disease pathways	Antidiabetic + Antihypertensive treatment	Combining different drugs to tackle various aspects of a disease.	[103,109]
Network Medicine	A systems-based approach that considers networks of genes, proteins, and diseases	Approaching multifactorial diseases with network models	Understanding diseases by modeling biological networks and their interactions.	[104]
Systems Pharmacology	Integrating systems biology with pharmacology to model drug effects	Modeling drug interactions with disease pathways	A comprehensive method that incorporates systems biology into pharmacology for better drug efficacy.	[106]
ADMET Profiling	Evaluation of Absorption, Distribution, Metabolism, Excretion, and Toxicity	Investigating ADMET properties of polypharmacological drugs	Analysis of drug properties to predict how the drug will interact with the body and its safety profile.	[106]
Bioinformatics	The use of computational tools to analyze biological data	Computational mapping for target identification	Utilizing computational methods to predict biological interactions and drug efficacy.	[104,110]
Drug-Target Networks	Visual representation of drug interactions with molecular targets	Identifying key drug-target interactions	Mapping drug interactions with various molecular targets to identify potential therapeutic applications.	[104,107]
Synergistic Drug Effects	A combination of drugs that work better together than separately	Anticancer agents work with other drugs to overcome resistance	Drugs that exhibit enhanced therapeutic effects when used in combination.	[103,110]
Chemoinformatics	The use of computational tools to analyze chemical data	Analysis of drug molecule structures and interactions	Analyzing the chemical properties of drugs to predict interactions and optimize drug design.	[106]
Pharmacokinetics	Study of drug absorption, distribution, metabolism, and excretion	Modeling how the body processes polypharmacological drugs	Analyzing how drugs are absorbed, distributed, metabolized, and excreted by the body to ensure safety.	[106,107].

**Table 4:** Representative Multi-Target Strategies in NDs.

Compound/ Strategy	Disease Context	Target Set	Mechanism	Evidence Level	BBB Consideration	Limitation	Citations
Donepezil-based hybrids	AD	AChE, BACE1, Aβ, oxidative stress	Cognitive + anti-amyloid effects	Mostly pre-clinical/early	BBB favorable for donepezil core	Hybrid safety uncertain	[122]
Ladostigil-type compounds	AD/PD models	MAO-A/B, cholinesterases, neuroprotection	Monoamine + cholinergic modulation	Preclinical/clinical exploration	CNS active	Limited disease-modifying proof	[123]
MitoQ/SS-31	AD/PD/ALS models	Mitochondrial ROS, bioenergetics	Reduces mitochondrial oxidative injury	Preclinical/clinical testing	Delivery and dose concerns	Translation inconsistent	[124]
Curcumin	AD/PD models	NF-κB, Nrf2, Aβ, inflammation	Anti-inflammatory/antioxidant	Preclinical; limited clinical	Poor bioavailability	Low potency, formulation issues	[125]
Resveratrol	AD/PD models	SIRT1, AMPK, inflammation	Metabolic and inflammatory modulation	Preclinical/clinical studies	Limited bioavailability	Mixed clinical outcomes	[126]
FAAH/MAGL modulation	AD/PD/MS models	ECS enzymes, inflammation, and excitotoxicity	Enhances endocannabinoid tone	Mainly pre-clinical	CNS activity varies	Psychoactivity/safety concerns	[127]
Hybrid molecular designs (tacrine-based)	AD/PD models	AChE, BACE1, oxidative stress, amyloid aggregation	Reduces amyloid aggregation, neuroprotective	Preclinical/clinical	BBB penetration varies	Safety, bioavailability concerns	[128]
Nanoparticle-guided drug delivery	AD/PD/HD	Multiple targets via nanomedicine	Targeted drug delivery to the brain	Ongoing preclinical/clinical	BBB penetration enhanced by nanocarriers	Delivery system complexity, dose regulation	[129]
Coumarin-based hybrids	AD/PD models	AChE, MAO-B, amyloid aggregation, neuroinflammation	Hybrid inhibitors targeting multiple AD-related mechanisms	Preclinical	BBB penetration varies	Low potency, formulation challenges	[125]
Polyphenols (Curcumin, Quercetin)	AD/PD models	Inflammation, oxidative stress, and amyloid aggregation	Antioxidant, anti-inflammatory, and metal chelation	Preclinical	Low bioavailability	Limited clinical validation	[130]

**Table 5:** Representative ECS-Based Polypharmacological Strategies in NDs.

Compound/Strategy	Disease Context	Target Set	Mechanism	Evidence Level	BBB Consideration	Limitation	Citations
Dual FAAH/MAGL Inhibition	AD, PD, MS	ECS enzymes, neuroinflammation, excitotoxicity, and	Enhances endocannabinoid tone, reduces oxidative stress, and neuroinflammation	Preclinical/clinical trials	CNS activity varies	Psychoactivity, receptor desensitization	[127]
CB1/CB2 Dual Agonists	MS, Parkinson’s Disease (PD)	CB1, CB2 receptors, neuroprotection, inflammation	Neuroprotective effects, immune modulation, and synaptic plasticity	Preclinical/clinical	CNS active	Limited clinical validation	[133]
NADA (N-arachidonoyldopamine)	Neurodegeneration models	CB1, neuroprotection	Protects neurons from excitotoxic damage, reduces microglial activation	Preclinical	CNS active	CB1 receptor desensitization, limited clinical data	[134]
Triple Target ECS Compounds	Neurodegeneration models	CB1, CB2, COX, TRPV1	Multi-target modulation of inflammation, oxidative stress, and neuroinflammation	Preclinical/clinical	CNS active	Dose regulation, efficacy variability	[129]
CB2 Receptor Agonists	Glioblastoma, MS, AD, PD	CB2 receptors, immune modulation, glioblastoma progression	Reduces tumor-associated macrophages, modulates immune response	Preclinical/clinical trials	CNS penetration varies	Limited clinical validation, receptor selectivity	[135]
Endocannabinoid Modulators	Stroke, TBI, AD, PD	CB1, CB2, oxidative stress, neuroinflammation	Modulates redox states, reduces inflammation, neuroprotection	Preclinical	CNS penetration varies	Low clinical translation, side effects	[136]

**Table 6:** Representative Multi-Target Modulation Strategies in NDs.

Compound/ Strategy	Disease Context	Target Set	Mechanism	Evidence Level	BBB Consideration	Limitation	Citations
Nrf2 activators	AD, PD, HD, ALS	Nrf2–Keap1 pathway, oxidative stress, inflammation	Enhances antioxidant response, cytoprotective signaling	Preclinical/clinical	CNS penetration varies	Limited clinical data on efficacy	[137]
NF-κB inhibitors	AD, PD, MS	NF-κB pathway, neuroinflammation	Reduces pro-inflammatory cytokine release	Preclinical/clinical	BBB favorable for some inhibitors	Safety concerns with chronic use	[138]
Curcumin hybrids	AD, PD	NF-κB, Nrf2, Aβ aggregation, inflammation	Anti-inflammatory, antioxidant, and amyloid plaque reduction	Preclinical/limited clinical	Poor bioavailability	Low potency, formulation issues	[125]
MitoQ	AD, PD, ALS	Mitochondrial ROS, mitochondrial dysfunction	Reduces mitochondrial oxidative injury	Preclinical/clinical	Limited CNS penetration	Inconsistent translation to clinical results	[124]
FAAH/MAGL dual inhibitors	AD, PD, MS	ECS enzymes, neuroinflammation, and excitotoxicity	Enhances endocannabinoid tone, neuroprotective signaling	Preclinical/clinical trials	CNS activity varies	Psychoactivity, receptor desensitization	[127]
TREM2/CSF1R modulators	AD, PD, MS	Microglial phenotype, immune signaling	Shifts microglia to a neuroprotective M2 phenotype	Preclinical/clinical trials	BBB penetration varies	Limited clinical validation	[135]
Resveratrol/Quercetin hybrids	AD, PD, HD	AMPK, SIRT1, inflammation, oxidative stress	Anti-inflammatory, antioxidant, metabolic modulation	Preclinical/clinical studies	Poor bioavailability	Low potency, mixed clinical outcomes	[126]
Dual CB1/CB2 agonists	PD, MS	CB1, CB2 receptors, neuroinflammation	Neuroprotective effects, immune modulation	Preclinical/clinical	CNS active	Limited clinical validation, receptor selectivity	[133]
TRPV1 channel modulators	AD, PD, HD	TRPV1, oxidative stress, inflammation	Modulates pain, inflammation, and synaptic plasticity	Preclinical/clinical	CNS penetration varies	Safety concerns with prolonged use	[136]

## Data Availability

No new data were created for this manuscript.

## References

[1] Pathak N., Vimal S.K., Tandon I., Agrawal L., Hongyi C., Bhattacharyya S. (2022). Neurodegenerative disorders of alzheimer, parkinsonism, amyotrophic lateral sclerosis and multiple sclerosis: An early diagnostic approach for precision treatment. Metab. Brain Dis..

[2] Gadhave D.G., Sugandhi V.V., Jha S.K., Nangare S.N., Gupta G., Singh S.K., Dua K., Cho H., Hansbro P.M., Paudel K.R. (2024). Neurodegenerative disorders: Mechanisms of degeneration and therapeutic approaches with their clinical relevance. Ageing Res. Rev..

[3] Qaed E., Aldahmash W., Mahyoub M.A., Al-Mutairi D.S., Tang Z., Almoiliqy M. (2025). Phosphocreatine Mitigates Doxorubicin-Induced Neurotoxicity in Rats by Regulating Mitochondrial Function and Apoptosis via the NF-κB/PGC-1α Pathway. NeuroMolecular Med..

[4] Jurcau A. (2021). Insights into the pathogenesis of neurodegenerative diseases: Focus on mitochondrial dysfunction and oxidative stress. Int. J. Mol. Sci..

[5] Sarkar C., Lipinski M.M. (2024). Role and function of peroxisomes in neuroinflammation. Cells.

[6] Roczkowsky A., Rachubinski R.A., Hobman T.C., Power C. (2025). Peroxisomes as emerging clinical targets in neuroinflammatory diseases. Front. Mol. Neurosci..

[7] Madireddy S., Madireddy S. (2023). Therapeutic strategies to ameliorate neuronal damage in epilepsy by regulating oxidative stress, mitochondrial dysfunction, and neuroinflammation. Brain Sci..

[8] Al-Hamadiny S., Salman R., Al-Rawe A. (2023). Nanocarriers and beyond: Innovations in overcoming barriers for effective CNS drug delivery. Trends Pharm. Biotechnol..

[9] Akhtar A., Andleeb A., Waris T.S., Bazzar M., Moradi A.-R., Awan N.R., Yar M. (2021). Neurodegenerative diseases and effective drug delivery: A review of challenges and novel therapeutics. J. Control. Release.

[10] Fang Y., Wang J., Zhao M., Zheng Q., Ren C., Wang Y., Zhang J. (2022). Progress and challenges in targeted protein degradation for neurodegenerative disease therapy. J. Med. Chem..

[11] Gribkoff V.K., Kaczmarek L.K. (2017). The need for new approaches in CNS drug discovery: Why drugs have failed, and what can be done to improve outcomes. Neuropharmacology.

[12] Lustoza Rodrigues T.C.M., de Sousa N.F., Dos Santos A.M.F., Aires Guimarães R.D., Scotti M.T., Scotti L. (2023). Challenges and discoveries in polypharmacology of neurodegenerative diseases. Curr. Top. Med. Chem..

[13] Kunwar O.K., Singh S. (2025). Neuroinflammation and neurodegeneration in Huntington’s disease: Genetic hallmarks, role of metals and organophosphates. Neurogenetics.

[14] Fasano M., Alberio T. (2023). Neurodegenerative disorders: From clinicopathology convergence to systems biology divergence. Handb. Clin. Neurol..

[15] Yue R., Dutta A. (2022). Computational systems biology in disease modeling and control, review and perspectives. npj Syst. Biol. Appl..

[16] Alqahtani T., Deore S.L., Kide A.A., Shende B.A., Sharma R., Chakole R.D., Nemade L.S., Kale N.K., Borah S., Deokar S.S. (2023). Mitochondrial dysfunction and oxidative stress in Alzheimer’s disease, and Parkinson’s disease, Huntington’s disease and amyotrophic lateral sclerosis-an updated review. Mitochondrion.

[17] Morató X., Pytel V., Jofresa S., Ruiz A., Boada M. (2022). Symptomatic and disease-modifying therapy pipeline for Alzheimer’s disease: Towards a personalized polypharmacology patient-centered approach. Int. J. Mol. Sci..

[18] Parikshak N.N., Gandal M.J., Geschwind D.H. (2015). Systems biology and gene networks in neurodevelopmental and neurodegenerative disorders. Nat. Rev. Genet..

[19] Vogel J.W., Corriveau-Lecavalier N., Franzmeier N., Pereira J.B., Brown J.A., Maass A., Botha H., Seeley W.W., Bassett D.S., Jones D.T. (2023). Connectome-based modelling of neurodegenerative diseases: Towards precision medicine and mechanistic insight. Nat. Rev. Neurosci..

[20] Haidar M.A., Ibeh S., Shakkour Z., Reslan M.A., Nwaiwu J., Moqidem Y.A., Sader G., Nickles R.G., Babale I., Jaffa A.A. (2022). Crosstalk between microglia and neurons in neurotrauma: An overview of the underlying mechanisms. Curr. Neuropharmacol..

[21] Cisneros J., Belton T.B., Shum G.C., Molakal C.G., Wong Y.C. (2022). Mitochondria-lysosome contact site dynamics and misregulation in neurodegenerative diseases. Trends Neurosci..

[22] Gupta S., You P., SenGupta T., Nilsen H., Sharma K. (2021). Crosstalk between different DNA repair pathways contributes to neurodegenerative diseases. Biology.

[23] Wang Z., Yang B. (2022). Strategies of polypharmacology. Polypharmacology: Principles and Methodologies.

[24] Albertini C., Salerno A., de Sena Murteira Pinheiro P., Bolognesi M.L. (2021). From combinations to multitarget-directed ligands: A continuum in Alzheimer’s disease polypharmacology. Med. Res. Rev..

[25] Gontijo V.S., Viegas F.P.D., Ortiz C.J., de Freitas Silva M., Damasio C.M., Rosa M.C., Campos T.G., Couto D.S., Tranches Dias K.S., Viegas C. (2020). Molecular hybridization as a tool in the design of multi-target directed drug candidates for neurodegenerative diseases. Curr. Neuropharmacol..

[26] Kooshki L., Zarneshan S.N., Fakhri S., Moradi S.Z., Echeverria J. (2023). The pivotal role of JAK/STAT and IRS/PI3K signaling pathways in neurodegenerative diseases: Mechanistic approaches to polyphenols and alkaloids. Phytomedicine.

[27] Nehmeh B., Rebehmed J., Nehmeh R., Taleb R., Akoury E. (2024). Unlocking therapeutic frontiers: Harnessing artificial intelligence in drug discovery for neurodegenerative diseases. Drug Discov. Today.

[28] Aldewachi H., Al-Zidan R.N., Conner M.T., Salman M.M. (2021). High-throughput screening platforms in the discovery of novel drugs for neurodegenerative diseases. Bioengineering.

[29] Snyder H. (2019). Literature review as a research methodology: An overview and guidelines. J. Bus. Res..

[30] Sukhera J. (2022). Narrative reviews in medical education: Key steps for researchers. J. Grad. Med. Educ..

[31] Snyder H. (2024). Designing the literature review for a strong contribution. J. Decis. Syst..

[32] Gregory A.T., Denniss A.R. (2018). An introduction to writing narrative and systematic reviews—Tasks, tips and traps for aspiring authors. Heart Lung Circ..

[33] Page M.J., McKenzie J.E., Bossuyt P.M., Boutron I., Hoffmann T.C., Mulrow C.D., Shamseer L., Tetzlaff J.M., Akl E.A., Brennan S.E. (2021). The PRISMA 2020 statement: An updated guideline for reporting systematic reviews. BMJ.

[34] Chopra G., Shabir S., Yousuf S., Kauts S., Bhat S.A., Mir A.H., Singh M.P. (2022). Proteinopathies: Deciphering physiology and mechanisms to develop effective therapies for neurodegenerative diseases. Mol. Neurobiol..

[35] Li Q., Babinchak W.M., Surewicz W.K. (2021). Cryo-EM structure of amyloid fibrils formed by the entire low complexity domain of TDP-43. Nat. Commun..

[36] Sanghai N., Tranmer G.K. (2023). Biochemical and molecular pathways in neurodegenerative diseases: An integrated view. Cells.

[37] Zhang L., Zheng Q., Zhang Z., Li H., Liu X., Sun J., Wang R. (2023). Application of metal–organic frameworks (MOFs) in environmental biosystems. Int. J. Mol. Sci..

[38] Sengupta U., Kayed R. (2022). Amyloid β, Tau, and α-Synuclein aggregates in the pathogenesis, prognosis, and therapeutics for neurodegenerative diseases. Prog. Neurobiol..

[39] Ren B., Zhang Y., Zhang M., Liu Y., Zhang D., Gong X., Feng Z., Tang J., Chang Y., Zheng J. (2019). Fundamentals of cross-seeding of amyloid proteins: An introduction. J. Mater. Chem. B.

[40] Wu Y., Ma B., Liu C., Li D., Sui G. (2024). Pathological involvement of protein phase separation and aggregation in neurodegenerative diseases. Int. J. Mol. Sci..

[41] Martiniakova M., Kovacova V., Mondockova V., Zemanova N., Babikova M., Biro R., Ciernikova S., Omelka R. (2023). Honey: A promising therapeutic supplement for the prevention and management of osteoporosis and breast cancer. Antioxidants.

[42] Anderson F.L., Biggs K.E., Rankin B.E., Havrda M.C. (2023). NLRP3 inflammasome in neurodegenerative disease. Transl. Res..

[43] Bathina S., Das U.N. (2023). Role of mitochondrial dysfunction in cellular lipid homeostasis and disease. Discov. Med..

[44] Lin M.-m., Liu N., Qin Z.-h., Wang Y. (2022). Mitochondrial-derived damage-associated molecular patterns amplify neuroinflammation in neurodegenerative diseases. Acta Pharmacol. Sin..

[45] Ho H.-J., Shirakawa H. (2022). Oxidative stress and mitochondrial dysfunction in chronic kidney disease. Cells.

[46] Ferreira J.P., Albuquerque H.M., Cardoso S.M., Silva A.M., Silva V.L. (2021). Dual-target compounds for Alzheimer’s disease: Natural and synthetic AChE and BACE-1 dual-inhibitors and their structure-activity relationship (SAR). Eur. J. Med. Chem..

[47] Whalley K. (2017). Improving opioids. Nat. Rev. Neurosci..

[48] Butler D., Bahr B.A. (2006). Oxidative stress and lysosomes: CNS-related consequences and implications for lysosomal enhancement strategies and induction of autophagy. Antioxid. Redox Signal..

[49] Lin M.G., Hurley J.H. (2016). Structure and function of the ULK1 complex in autophagy. Curr. Opin. Cell Biol..

[50] Lu Y., Li A., Liu A., Li M., Wang M. (2026). Autophagy in Cancer: Context-Dependent Regulation and Precision Nanomedicine-Enabled Therapeutic Targeting. Biomedicines.

[51] Davies H., Glodzik D., Morganella S., Yates L.R., Staaf J., Zou X., Ramakrishna M., Martin S., Boyault S., Sieuwerts A.M. (2017). HRDetect is a predictor of BRCA1 and BRCA2 deficiency based on mutational signatures. Nat. Med..

[52] Tang B.L. (2019). Why is NMNAT protective against neuronal cell death and axon degeneration, but inhibitory of axon regeneration?. Cells.

[53] Perdigão C., Barata M.A., Araújo M.N., Mirfakhar F.S., Castanheira J., Guimas Almeida C. (2020). Intracellular trafficking mechanisms of synaptic dysfunction in Alzheimer’s disease. Front. Cell. Neurosci..

[54] Ng B., White C.C., Klein H.-U., Sieberts S.K., McCabe C., Patrick E., Xu J., Yu L., Gaiteri C., Bennett D.A. (2017). An xQTL map integrates the genetic architecture of the human brain’s transcriptome and epigenome. Nat. Neurosci..

[55] Mordelt A., de Witte L.D. (2023). Microglia-mediated synaptic pruning as a key deficit in neurodevelopmental disorders: Hype or hope?. Curr. Opin. Neurobiol..

[56] Nilsson J., Cousins K.A., Gobom J., Portelius E., Chen-Plotkin A., Shaw L.M., Grossman M., Irwin D.J., Trojanowski J.Q., Zetterberg H. (2023). Cerebrospinal fluid biomarker panel of synaptic dysfunction in Alzheimer’s disease and other neurodegenerative disorders. Alzheimer’s Dement..

[57] Cai Q., Tammineni P. (2016). Alterations in mitochondrial quality control in Alzheimer’s disease. Front. Cell. Neurosci..

[58] Schneider P., Walters W.P., Plowright A.T., Sieroka N., Listgarten J., Goodnow R.A., Fisher J., Jansen J.M., Duca J.S., Rush T.S. (2020). Rethinking drug design in the artificial intelligence era. Nat. Rev. Drug Discov..

[59] Fallini C., Bassell G.J., Rossoll W. (2012). The ALS disease protein TDP-43 is actively transported in motor neuron axons and regulates axon outgrowth. Hum. Mol. Genet..

[60] Luna-Marco C., Iannantuoni F., Hermo-Argibay A., Devos D., Salazar J.D., Víctor V.M., Rovira-Llopis S. (2024). Cardiovascular benefits of SGLT2 inhibitors and GLP-1 receptor agonists through effects on mitochondrial function and oxidative stress. Free Radic. Biol. Med..

[61] Martinez M.A. (2022). Efficacy of repurposed antiviral drugs: Lessons from COVID-19. Drug Discov. Today.

[62] Kim H.J., Chung J.-W., Bang O.Y., Cho Y.H., Lim Y.J., Hwang J., Seo W.-K., Kim G.-M., Kim H.-J., Ahn M.-J. (2021). The role of factor Xa-independent pathway and anticoagulant therapies in cancer-related stroke. J. Clin. Med..

[63] Saleem S. (2021). Apoptosis, autophagy, necrosis and their multi galore crosstalk in neurodegeneration. Neuroscience.

[64] Jassim A.H., Inman D.M., Mitchell C.H. (2021). Crosstalk between dysfunctional mitochondria and inflammation in glaucomatous neurodegeneration. Front. Pharmacol..

[65] Baev A.Y., Vinokurov A.Y., Novikova I.N., Dremin V.V., Potapova E.V., Abramov A.Y. (2022). Interaction of mitochondrial calcium and ROS in neurodegeneration. Cells.

[66] Michalska P., León R. (2020). When it comes to an end: Oxidative stress crosstalk with protein aggregation and neuroinflammation induce neurodegeneration. Antioxidants.

[67] Xu X., Pang Y., Fan X. (2025). Mitochondria in oxidative stress, inflammation and aging: From mechanisms to therapeutic advances. Signal Transduct. Target. Ther..

[68] Stojanovic B., Milivojcevic Bevc I., Dimitrijevic Stojanovic M., Stojanovic B.S., Lazarevic T., Spasic M., Petrovic M., Stefanovic I., Markovic M., Nesic J. (2025). Oxidative stress, inflammation, and cellular senescence in neuropathic pain: Mechanistic crosstalk. Antioxidants.

[69] Jo D.S., Park N.Y., Cho D.-H. (2020). Peroxisome quality control and dysregulated lipid metabolism in neurodegenerative diseases. Exp. Mol. Med..

[70] Fransen M., Revenco I., Li H., Costa C.F., Lismont C., Van Veldhoven P.P. (2021). Peroxisomal dysfunction and oxidative stress in neurodegenerative disease: A bidirectional crosstalk. Peroxisome Biology: Experimental Models, Peroxisomal Disorders and Neurological Diseases.

[71] Nixon R.A., Rubinsztein D.C. (2024). Mechanisms of autophagy–lysosome dysfunction in neurodegenerative diseases. Nat. Rev. Mol. Cell Biol..

[72] Monaco A., Fraldi A. (2020). Protein aggregation and dysfunction of autophagy-lysosomal pathway: A vicious cycle in lysosomal storage diseases. Front. Mol. Neurosci..

[73] Ratti A., Buratti E. (2016). Physiological functions and pathobiology of TDP-43 and FUS/TLS proteins. J. Neurochem..

[74] Nadh A.G., Chakraborty S., Raju R., Rizvi A., Chaudhary R.K. (2025). Neuroinflammation and Autophagy in Neurodegenerative Therapy. Neuroinflammation and Autophagy in Neurodegeneration: Mechanistic Insights into Cellular Biology and Advanced Multimodal Therapeutics.

[75] Chauhan N.B. (2014). Chronic neurodegenerative consequences of traumatic brain injury. Restor. Neurol. Neurosci..

[76] Lepeta K., Lourenco M.V., Schweitzer B.C., Martino Adami P.V., Banerjee P., Catuara-Solarz S., de La Fuente Revenga M., Guillem A.M., Haidar M., Ijomone O.M. (2016). Synaptopathies: Synaptic dysfunction in neurological disorders–A review from students to students. J. Neurochem..

[77] Levin S., Godukhin O. (2017). Modulating effect of cytokines on mechanisms of synaptic plasticity in the brain. Biochemistry.

[78] Alexandersen C.G., Brennan G.S., Brynildsen J.K., Henderson M.X., Iturria-Medina Y., Bassett D.S. (2026). Network Models of Neurodegeneration: Bridging Neuronal Dynamics and Disease Progression. IEEE Rev. Biomed. Eng..

[79] Doroszkiewicz J., Mroczko J., Winkel I., Mroczko B. (2024). Metabolic and immune system dysregulation: Unraveling the connections between Alzheimer’s disease, diabetes, inflammatory bowel diseases, and rheumatoid arthritis. J. Clin. Med..

[80] Castro-Gomez S., Heneka M.T. (2024). Innate immune activation in neurodegenerative diseases. Immunity.

[81] Zhang W., Xiao D., Mao Q., Xia H. (2023). Role of neuroinflammation in neurodegeneration development. Signal Transduct. Target. Ther..

[82] Weiner H.L. (2025). Immune mechanisms and shared immune targets in neurodegenerative diseases. Nat. Rev. Neurol..

[83] Sarazin M., Lagarde J., El Haddad I., de Souza L.C., Bellier B., Potier M.-C., Bottlaender M., Dorothee G. (2024). The path to next-generation disease-modifying immunomodulatory combination therapies in Alzheimer’s disease. Nat. Aging.

[84] Sidiropoulou G.A., Metaxas A., Kourti M. (2023). Natural antioxidants that act against Alzheimer’s disease through modulation of the NRF2 pathway: A focus on their molecular mechanisms of action. Front. Endocrinol..

[85] Sawant N., Morton H., Kshirsagar S., Reddy A.P., Reddy P.H. (2021). Mitochondrial abnormalities and synaptic damage in Huntington’s disease: A focus on defective mitophagy and mitochondria-targeted therapeutics. Mol. Neurobiol..

[86] Abbassi Y., Fink D., Cei F., Niccolai E., Amedei A. (2026). TDP-43–immunity–microbiota axis in amyotrophic lateral sclerosis: A potential pathogenic mechanism. Neural Regen. Res..

[87] Wingo T.S., Liu Y., Gerasimov E.S., Vattathil S.M., Wynne M.E., Liu J., Lori A., Faundez V., Bennett D.A., Seyfried N.T. (2022). Shared mechanisms across the major psychiatric and neurodegenerative diseases. Nat. Commun..

[88] Li P., Nie Y., Yu J. (2015). An effective method to identify shared pathways and common factors among neurodegenerative diseases. PLoS ONE.

[89] Butler R., Bradford D., Rodgers K.E. (2022). Analysis of shared underlying mechanism in neurodegenerative disease. Front. Aging Neurosci..

[90] Costa Sa A.C., Madsen H., Brown J.R. (2019). Shared molecular signatures across neurodegenerative diseases and herpes virus infections highlights potential mechanisms for maladaptive innate immune responses. Sci. Rep..

[91] Shvetcov A., Johnson E.C., Winchester L.M., Walker K.A., Wilkins H.M., Thompson T.G., Rothstein J.D., Krish V., Imam F.B., Consortium G.N.P. (2025). APOE ε4 carriers share immune-related proteomic changes across neurodegenerative diseases. Nat. Med..

[92] Birmann P.T., Casaril A.M., Abenante L., Penteado F., Brüning C.A., Savegnago L., SNM E.L. (2023). Neuropharmacology of organoselenium compounds in mental disorders and degenerative diseases. Curr. Med. Chem..

[93] Aharoni R. (2014). Immunomodulation neuroprotection and remyelination—The fundamental therapeutic effects of glatiramer acetate: A critical review. J. Autoimmun..

[94] Ochneva A., Zorkina Y., Abramova O., Pavlova O., Ushakova V., Morozova A., Zubkov E., Pavlov K., Gurina O., Chekhonin V. (2022). Protein misfolding and aggregation in the brain: Common pathogenetic pathways in neurodegenerative and mental disorders. Int. J. Mol. Sci..

[95] Kaur D., Sharma V., Deshmukh R. (2019). Activation of microglia and astrocytes: A roadway to neuroinflammation and Alzheimer’s disease. Inflammopharmacology.

[96] Ma Y., Song R., Duan C. (2025). Mitochondrial quality control and transfer communication in neurological disorders and neuroinflammation. Front. Immunol..

[97] Li H., Cai R., Zhou Y., Jiang Y., Tan S. (2025). cGAS-STING signaling in brain aging and neurodegeneration: Molecular links and therapeutic perspectives. J. Neuroinflamm..

[98] Zhou X., Wang J., Yu L., Qiao G., Qin D., Law B.Y.-K., Ren F., Wu J., Wu A. (2024). Mitophagy and cGAS–STING crosstalk in neuroinflammation. Acta Pharm. Sin. B.

[99] Yang R., Wang R., Xu A., Zhang J., Ma J. (2024). Mitigating neurodegenerative diseases: The protective influence of baicalin and baicalein through neuroinflammation regulation. Front. Pharmacol..

[100] Liu W., Taso O., Wang R., Bayram S., Graham A.C., Garcia-Reitboeck P., Mallach A., Andrews W.D., Piers T.M., Botia J.A. (2020). Trem2 promotes anti-inflammatory responses in microglia and is suppressed under pro-inflammatory conditions. Hum. Mol. Genet..

[101] Fan X., Diao W., Wang H., Yin X., Qian W. (2025). Interferon regulatory factors as a potential therapeutic target for neuroinflammation: A focus on Alzheimer’s disease. Int. J. Mol. Sci..

[102] Song T.-T., Cai R.-S., Hu R., Xu Y.-S., Qi B.-N., Xiong Y.-A. (2021). The important role of TFEB in autophagy-lysosomal pathway and autophagy-related diseases: A systematic review. Eur. Rev. Med. Pharmacol. Sci..

[103] Ryszkiewicz P., Malinowska B., Schlicker E. (2023). Polypharmacology: Promises and new drugs in 2022. Pharmacol. Rep..

[104] Ravikumar B., Aittokallio T. (2018). Improving the efficacy-safety balance of polypharmacology in multi-target drug discovery. Expert Opin. Drug Discov..

[105] Zhao S., Iyengar R. (2012). Systems pharmacology: Network analysis to identify multiscale mechanisms of drug action. Annu. Rev. Pharmacol. Toxicol..

[106] Abdelsayed M. (2025). AI-driven polypharmacology in small-molecule drug discovery. Int. J. Mol. Sci..

[107] Tomaselli D., Lucidi A., Rotili D., Mai A. (2020). Epigenetic polypharmacology: A new frontier for epi-drug discovery. Med. Res. Rev..

[108] Reddy A.S., Zhang S. (2013). Polypharmacology: Drug discovery for the future. Expert Rev. Clin. Pharmacol..

[109] Ciceri P., Müller S., O’mahony A., Fedorov O., Filippakopoulos P., Hunt J.P., Lasater E.A., Pallares G., Picaud S., Wells C. (2014). Dual kinase-bromodomain inhibitors for rationally designed polypharmacology. Nat. Chem. Biol..

[110] Knight Z.A., Lin H., Shokat K.M. (2010). Targeting the cancer kinome through polypharmacology. Nat. Rev. Cancer.

[111] Arrué L., Cigna-Méndez A., Barbosa T., Borrego-Muñoz P., Struve-Villalobos S., Oviedo V., Martínez-García C., Sepúlveda-Lara A., Millán N., Márquez Montesinos J.C. (2022). New drug design avenues targeting Alzheimer’s disease by pharmacoinformatics-aided tools. Pharmaceutics.

[112] Zimmermann G.R., Lehar J., Keith C.T. (2007). Multi-target therapeutics: When the whole is greater than the sum of the parts. Drug Discov. Today.

[113] Xiao X., Yan X., Liang C., Yang Y. (2026). Metabolic dysfunction and mitochondrial failure in Alzheimer’s disease: Integrating pathophysiology, clinical evidence and emerging interventions. Front. Neurol..

[114] O’Brien K., Blair P. (2021). Endocannabinoid system. Medicinal Cannabis and CBD in Mental Healthcare.

[115] Papa A., Pasquini S., Contri C., Gemma S., Campiani G., Butini S., Varani K., Vincenzi F. (2022). Polypharmacological approaches for CNS diseases: Focus on endocannabinoid degradation inhibition. Cells.

[116] Stefan S.M., Rafehi M. (2024). Medicinal polypharmacology—A scientific glossary of terminology and concepts. Front. Pharmacol..

[117] Jia J., Zhu F., Ma X., Cao Z.W., Li Y.X., Chen Y.Z. (2009). Mechanisms of drug combinations: Interaction and network perspectives. Nat. Rev. Drug Discov..

[118] Li J., Lu C., Jiang M., Niu X., Guo H., Li L., Bian Z., Lin N., Lu A. (2012). Traditional chinese medicine-based network pharmacology could lead to new multicompound drug discovery. Evid. Based Complement. Altern. Med..

[119] Seghetti F., Gobbi S., Belluti F., Rampa A., Bisi A. (2022). Interplay between endocannabinoid system and neurodegeneration: Focus on Polypharmacology. Curr. Med. Chem..

[120] Ligresti A., De Petrocellis L., Di Marzo V. (2016). From phytocannabinoids to cannabinoid receptors and endocannabinoids: Pleiotropic physiological and pathological roles through complex pharmacology. Physiol. Rev..

[121] Stasiulewicz A., Znajdek K., Grudzień M., Pawiński T., Sulkowska J.I. (2020). A guide to targeting the endocannabinoid system in drug design. Int. J. Mol. Sci..

[122] Angelova V.T., Stoyanov B.P., Simeonova R. (2024). New insights into the development of donepezil-based hybrid and natural molecules as multi-target drug agents for Alzheimer’s disease treatment. Molecules.

[123] Agis-Torres A., Sollhuber M., Fernandez M., Sanchez-Montero J. (2014). Multi-target-directed ligands and other therapeutic strategies in the search of a real solution for Alzheimer’s disease. Curr. Neuropharmacol..

[124] Cacabelos R., Martínez-Iglesias O., Cacabelos N., Carrera I., Corzo L., Naidoo V. (2024). Therapeutic options in Alzheimer’s disease: From classic acetylcholinesterase inhibitors to multi-target drugs with pleiotropic activity. Life.

[125] Rullo M., La Spada G., Stefanachi A., Macchia E., Pisani L., Leonetti F. (2025). Playing Around the Coumarin Core in the Discovery of Multimodal Compounds Directed at Alzheimer’s-Related Targets: A Recent Literature Overview. Molecules.

[126] Alarcón-Espósito J., Mallea M., Rodríguez-Lavado J. (2021). From hybrids to new scaffolds: The latest medicinal chemistry goals in multi-target directed ligands for Alzheimer’s disease. Curr. Neuropharmacol..

[127] Vahid Z.F., Eskandani M., Dadashi H., Vandghanooni S., Rashidi M.-R. (2024). Recent advances in potential enzymes and their therapeutic inhibitors for the treatment of Alzheimer’s disease. Heliyon.

[128] Simunkova M., Alwasel S.H., Alhazza I.M., Jomova K., Kollar V., Rusko M., Valko M. (2019). Management of oxidative stress and other pathologies in Alzheimer’s disease. Arch. Toxicol..

[129] Riccardi C., Napolitano F., Montesarchio D., Sampaolo S., Melone M.A.B. (2021). Nanoparticle-guided brain drug delivery: Expanding the therapeutic approach to neurodegenerative diseases. Pharmaceutics.

[130] Dhakal S., Kushairi N., Phan C.W., Adhikari B., Sabaratnam V., Macreadie I. (2019). Dietary polyphenols: A multifactorial strategy to target Alzheimer’s disease. Int. J. Mol. Sci..

[131] Coles M., Steiner-Lim G.Z., Karl T. (2022). Therapeutic properties of multi-cannabinoid treatment strategies for Alzheimer’s disease. Front. Neurosci..

[132] Fernández-Ruiz J. (2019). The biomedical challenge of neurodegenerative disorders: An opportunity for cannabinoid-based therapies to improve on the poor current therapeutic outcomes. Br. J. Pharmacol..

[133] Green H.M., Manning J.J., Greig I.R., Ross R.A., Finlay D.B., Glass M. (2024). Positive allosteric modulation of the cannabinoid CB1 receptor potentiates endocannabinoid signalling and changes ERK1/2 phosphorylation kinetics. Br. J. Pharmacol..

[134] Grabiec U., Koch M., Kallendrusch S., Kraft R., Hill K., Merkwitz C., Ghadban C., Lutz B., Straiker A., Dehghani F. (2012). The endocannabinoid N-arachidonoyldopamine (NADA) exerts neuroprotective effects after excitotoxic neuronal damage via cannabinoid receptor 1 (CB1). Neuropharmacology.

[135] Duan J., Chen J., Lin Y., Lin S.L., Wu J. (2024). Endocannabinoid receptor 2 function is associated with tumor-associated macrophage accumulation and increases in T cell number to initiate a potent antitumor response in a syngeneic murine model of glioblastoma. Cannabis Cannabinoid Res..

[136] Paloczi J., Varga Z.V., Hasko G., Pacher P. (2018). Neuroprotection in oxidative stress-related neurodegenerative diseases: Role of endocannabinoid system modulation. Antioxid. Redox Signal..

[137] Kitamura H., Oishi T., Murakami S., Yamada-Kato T., Okunishi I., Yamamoto M., Katori Y., Motohashi H. (2022). Establishment of Neh2-Cre: TdTomato reporter mouse for monitoring the exposure history to electrophilic stress. Free Radic. Biol. Med..

[138] Singh S., Singh T.G. (2020). Role of nuclear factor kappa B (NF-κB) signalling in neurodegenerative diseases: An mechanistic approach. Curr. Neuropharmacol..

[139] Esmaeili Y., Yarjanli Z., Pakniya F., Bidram E., Łos M.J., Eshraghi M., Klionsky D.J., Ghavami S., Zarrabi A. (2022). Targeting autophagy, oxidative stress, and ER stress for neurodegenerative disease treatment. J. Control. Release.

[140] Pant A., Dasgupta D., Tripathi A., Pyaram K. (2023). Beyond antioxidation: Keap1–Nrf2 in the development and effector functions of adaptive immune cells. Immunohorizons.

[141] Dhapola R., Paidlewar M., Kumari S., Sharma P., Vellingiri B., Medhi B., HariKrishnaReddy D. (2025). cGAS-STING and neurodegenerative diseases: A molecular crosstalk and therapeutic perspective. Int. Immunopharmacol..

[142] Shah A.J., Mir P.A., Adnan M., Patel M., Maqbool M., Mir R.H., Masoodi M.H. (2023). Synthetic and Natural Bioactive Molecules in Balancing the Crosstalk among Common Signaling Pathways in Alzheimer’s Disease: Understanding the Neurotoxic Mechanisms for Therapeutic Intervention. ACS Omega.

[143] Pathak C., Kabra U.D. (2024). A comprehensive review of multi-target directed ligands in the treatment of Alzheimer’s disease. Bioorganic Chem..

[144] Bajda M., Guzior N., Ignasik M., Malawska B. (2011). Multi-target-directed ligands in Alzheimer’s disease treatment. Curr. Med. Chem..

[145] Hossain M.S., Hussain M.H. (2025). Multi-Target Drug Design in Alzheimer’s Disease Treatment: Emerging Technologies, Advantages, Challenges, and Limitations. Pharmacol. Res. Perspect..

[146] Nahar L., Charoensup R., Kalieva K., Habibi E., Guo M., Wang D., Kvasnica M., Onder A., Sarker S. (2025). Natural products in neurodegenerative diseases: Recent advances and future outlook. Front. Pharmacol..

[147] Cui D., Chen Y., Ye B., Guo W., Wang D., He J. (2023). Natural products for the treatment of neurodegenerative diseases. Phytomedicine.

[148] Goyal R., Mittal P., Gautam R.K., Kamal M.A., Perveen A., Garg V., Alexiou A., Saboor M., Haque S., Farhana A. (2024). Natural products in the management of neurodegenerative diseases. Nutr. Metab..

[149] Geldenhuys W.J., van der Schyf C.J. (2013). Rationally designed multi-targeted agents against neurodegenerative diseases. Curr. Med. Chem..

[150] Saraiva C., Praça C., Ferreira R., Santos T., Ferreira L., Bernardino L. (2016). Nanoparticle-mediated brain drug delivery: Overcoming blood–brain barrier to treat neurodegenerative diseases. J. Control. Release.

[151] Bhal S., Kundu C.N. (2023). Targeting crosstalk of signaling pathways in cancer stem cells: A promising approach for development of novel anti-cancer therapeutics. Med. Oncol..

[152] Bilawal A., Shahab M., Shah Z., Ishfaq M. (2025). Integrated network analysis in pharmacology: Decoding interactions and pathways for therapeutic insights. Computational Methods in Medicinal Chemistry, Pharmacology, and Toxicology.

[153] Thombre K.R., Gabhane V., Gupta K.R., Umekar M.J. (2026). Network pharmacology in the multi-omics era: Uncovering novel therapeutic strategies. Discov. Chem..

[154] Joshi C.P., Baldi A., Kumar N., Pradhan J. (2025). Harnessing network pharmacology in drug discovery: An integrated approach. Naunyn-Schmiedeberg’s Arch. Pharmacol..

[155] Alkhammash A., Alotaibi G., Algethami A., Alqarni M. (2025). Decoding apoptosis-associated pathways in inflammatory and neurodegenerative diseases: A network pharmacology based drug discovery approach. Eur. J. Pharmacol..

[156] Akgüller Ö., Balcı M.A., Cioca G. (2025). A Multi-Modal Graph Neural Network Framework for Parkinson’s Disease Therapeutic Discovery. Int. J. Mol. Sci..

[157] Hopkins A.L. (2008). Network pharmacology: The next paradigm in drug discovery. Nat. Chem. Biol..

[158] Berger S.I., Iyengar R. (2009). Network analyses in systems pharmacology. Bioinformatics.

[159] Zhao L., Zhang H., Li N., Chen J., Xu H., Wang Y., Liang Q. (2023). Network pharmacology, a promising approach to reveal the pharmacology mechanism of Chinese medicine formula. J. Ethnopharmacol..

[160] Arena C., Gado F., Mannelli L.D.C., Cervetto C., Carpi S., Reynoso-Moreno I., Polini B., Vallini E., Chicca S., Lucarini E. (2020). The endocannabinoid system dual-target ligand N-cycloheptyl-1, 2-dihydro-5-bromo-1-(4-fluorobenzyl)-6-methyl-2-oxo-pyridine-3-carboxamide improves disease severity in a mouse model of multiple sclerosis. Eur. J. Med. Chem..

[161] Chen Q., Chen G., Wang Q. (2025). Application of network pharmacology in the treatment of neurodegenerative diseases with traditional Chinese medicine. Planta Medica.

[162] Kibble M., Saarinen N., Tang J., Wennerberg K., Mäkelä S., Aittokallio T. (2015). Network pharmacology applications to map the unexplored target space and therapeutic potential of natural products. Nat. Prod. Rep..

[163] Shahin R., Jaafreh S., Azzam Y. (2025). Tracking protein kinase targeting advances: Integrating QSAR into machine learning for kinase-targeted drug discovery. Future Sci. OA.

[164] Sun D., Chen P., Tao L., Ma P., Meng L., Yin S., Zhang B., Li S. (2026). Network Pharmacology in Food-Medicine Homology: AI-Driven Decoding of Multi-Target Synergy from Molecular Networks to Precision Health. Acupunct. Herb. Med..

[165] Boezio B., Audouze K., Ducrot P., Taboureau O. (2017). Network-based approaches in pharmacology. Mol. Inform..

[166] Muhammad J., Khan A., Ali A., Fang L., Yanjing W., Xu Q., Wei D.-Q. (2018). Network pharmacology: Exploring the resources and methodologies. Curr. Top. Med. Chem..

[167] Li S., Zhang B. (2013). Traditional Chinese medicine network pharmacology: Theory, methodology and application. Chin. J. Nat. Med..

[168] Wang W., Wang S., Liu T., Ma Y., Huang S., Lei L., Wen A., Ding Y. (2020). Resveratrol: Multi-targets mechanism on neurodegenerative diseases based on network pharmacology. Front. Pharmacol..

[169] Hang Z., Zhou L., Xing C., Wen Y., Du H. (2023). The blood-brain barrier, a key bridge to treat neurodegenerative diseases. Ageing Res. Rev..

[170] Nogales C., Mamdouh Z.M., List M., Kiel C., Casas A.I., Schmidt H.H. (2022). Network pharmacology: Curing causal mechanisms instead of treating symptoms. Trends Pharmacol. Sci..

[171] Noor F., Asif M., Ashfaq U.A., Qasim M., Tahir ul Qamar M. (2023). Machine learning for synergistic network pharmacology: A comprehensive overview. Brief. Bioinform..

[172] Zhang K., Yang X., Wang Y., Yu Y., Huang N., Li G., Li X., Wu J.C., Yang S. (2025). Artificial intelligence in drug development. Nat. Med..

[173] Jiang W., Ye W., Tan X., Bao Y.-J. (2025). Network-based multi-omics integrative analysis methods in drug discovery: A systematic review. BioData Min..

[174] Noor F., Tahir ul Qamar M., Ashfaq U.A., Albutti A., Alwashmi A.S., Aljasir M.A. (2022). Network pharmacology approach for medicinal plants: Review and assessment. Pharmaceuticals.

[175] Liu L., Zhu Y., Fu P., Yang J. (2022). A network pharmacology based research on the mechanism of donepezil in treating alzheimer’s disease. Front. Aging Neurosci..

[176] Liu Y., Zhang S., Liu K., Hu X., Gu X. (2024). Advances in drug discovery based on network pharmacology and omics technology. Curr. Pharm. Anal..

[177] Akki A.J., Patil S.A., Hungund S., Sahana R., Patil M.M., Kulkarni R.V., Reddy K.R., Zameer F., Raghu A.V. (2024). Advances in Parkinson’s disease research–a computational network pharmacological approach. Int. Immunopharmacol..

[178] Meng Q., Liu Q., Mi Y., Xu L., Wang F., Mu D., Liu Y., Yang Y., Huang Y., He D. (2025). Multi-dimensional data-driven computational drug repurposing strategy for screening novel neuroprotective agents in ischemic stroke. Theranostics.

[179] Lee W.-Y., Park K.-I., Bak S.-B., Lee S., Bae S.-J., Kim M.-J., Park S.-D., Kim C.O., Kim J.-H., Kim Y.W. (2024). Evaluating current status of network pharmacology for herbal medicine focusing on identifying mechanisms and therapeutic effects. J. Adv. Res..

[180] Pandey A.K., Ghiassian S.D., Loscalzo J. (2026). Network-based precision medicine and systems pharmacology. Br. J. Pharmacol..

[181] Ali H. (2023). Artificial intelligence in multi-omics data integration: Advancing precision medicine, biomarker discovery and genomic-driven disease interventions. Int. J. Sci. Res. Arch..

[182] Hessler G., Baringhaus K.-H. (2018). Artificial intelligence in drug design. Molecules.

[183] Serrano D.R., Luciano F.C., Anaya B.J., Ongoren B., Kara A., Molina G., Ramirez B.I., Sánchez-Guirales S.A., Simon J.A., Tomietto G. (2024). Artificial intelligence (AI) applications in drug discovery and drug delivery: Revolutionizing personalized medicine. Pharmaceutics.

[184] Karczewski K.J., Snyder M.P. (2018). Integrative omics for health and disease. Nat. Rev. Genet..

[185] Hasin Y., Seldin M., Lusis A. (2017). Multi-omics approaches to disease. Genome Biol..

[186] Baysoy A., Bai Z., Satija R., Fan R. (2023). The technological landscape and applications of single-cell multi-omics. Nat. Rev. Mol. Cell Biol..

[187] Gebreyesus S.T., Siyal A.A., Kitata R.B., Chen E.S.-W., Enkhbayar B., Angata T., Lin K.-I., Chen Y.-J., Tu H.-L. (2022). Streamlined single-cell proteomics by an integrated microfluidic chip and data-independent acquisition mass spectrometry. Nat. Commun..

[188] Ding S., Khan A.I., Cai X., Song Y., Lyu Z., Du D., Dutta P., Lin Y. (2020). Overcoming blood–brain barrier transport: Advances in nanoparticle-based drug delivery strategies. Mater. Today.

[189] Volceanov A. (2022). Blood-Brain Delivery Methods Using Nanotechnology. Pharmaceutics.

[190] Rafati N., Zarepour A., Bigham A., Khosravi A., Naderi-Manesh H., Iravani S., Zarrabi A. (2024). Nanosystems for targeted drug Delivery: Innovations and challenges in overcoming the Blood-Brain barrier for neurodegenerative disease and cancer therapy. Int. J. Pharm..

[191] Goel F., Kumar D., Singh P., Rai S.N., Yadav D.K. (2026). Molecular crosstalk between miRNAs and lncRNAs in neurodegenerative disease pathways. Mol. Biol. Rep..

[192] Yuasa-Kawada J., Kinoshita-Kawada M., Hiramoto M., Yamagishi S., Mishima T., Yasunaga S., Tsuboi Y., Hattori N., Wu J.Y. (2026). Neuronal guidance signaling in neurodegenerative diseases: Key regulators that function at neuron-glia and neuroimmune interfaces. Neural Regen. Res..

[193] Carata E., Destino M., Tenuzzo B.A., Panzarini E. (2025). Inter-Organ Crosstalk in Neurodegenerative Disease. Life.

[194] Wilson D.M., Cookson M.R., Van Den Bosch L., Zetterberg H., Holtzman D.M., Dewachter I. (2023). Hallmarks of neurodegenerative diseases. Cell.

[195] Yao L., Chen R., Zheng Z., Hatami M., Koc S., Wang X., Bai Y., Yao C., Lu G., Skutella T. (2025). Translational evaluation of metabolic risk factors impacting DBS efficacy for PD-related sleep and depressive disorders: Preclinical, prospective and cohort studies. Int. J. Surg..

[196] Katsoulaki E.-E., Dimopoulos D., Hadjipavlou-Litina D. (2025). Multitarget compounds designed for Alzheimer, Parkinson, and Huntington neurodegeneration diseases. Pharmaceuticals.

[197] Ju Y., Tam K.Y. (2022). Pathological mechanisms and therapeutic strategies for Alzheimer’s disease. Neural Regen. Res..

[198] Cavalli A., Bolognesi M.L., Minarini A., Rosini M., Tumiatti V., Recanatini M., Melchiorre C. (2008). Multi-target-directed ligands to combat neurodegenerative diseases. J. Med. Chem..

[199] Sweeney M.D., Sagare A.P., Zlokovic B.V. (2018). Blood–brain barrier breakdown in Alzheimer disease and other neurodegenerative disorders. Nat. Rev. Neurol..

[200] Mead R.J., Shan N., Reiser H.J., Marshall F., Shaw P.J. (2023). Amyotrophic lateral sclerosis: A neurodegenerative disorder poised for successful therapeutic translation. Nat. Rev. Drug Discov..

[201] Rosser A.E., Busse M.E., Gray W.P., Badin R.A., Perrier A.L., Wheelock V., Cozzi E., Martin U.P., Salado-Manzano C., Mills L.J. (2022). Translating cell therapies for neurodegenerative diseases: Huntington’s disease as a model disorder. Brain.

[202] Shaikh N.K. (2026). Synergistic Approach and the Treatment of Neurodegenerative Disorder. Therapeutic Potential of Natural Products in Neurodegenerative Disorders.

[203] Ashok A., Andrabi S.S., Mansoor S., Kuang Y., Kwon B.K., Labhasetwar V. (2022). Antioxidant therapy in oxidative stress-induced neurodegenerative diseases: Role of nanoparticle-based drug delivery systems in clinical translation. Antioxidants.

[204] Stanić Z. (2017). Curcumin, a compound from natural sources, a true scientific challenge–A review. Plant Foods Hum. Nutr..

[205] Aizpurua-Olaizola O., Elezgarai I., Rico-Barrio I., Zarandona I., Etxebarria N., Usobiaga A. (2017). Targeting the endocannabinoid system: Future therapeutic strategies. Drug Discov. Today.

[206] Singh K., Gupta J.K., Lakhchora G., Jain D., Bhatt A., Sharma M.C., Chaitanya M., Tabish M. (2025). Advance Nanotechnology-Based Drug Delivery Systems for Alzheimer’s Disease: Advancements and Future Perspectives. Curr. Alzheimer Res..

[207] Strafella C., Caputo V., Galota M.R., Zampatti S., Marella G., Mauriello S., Cascella R., Giardina E. (2018). Application of precision medicine in neurodegenerative diseases. Front. Neurol..

[208] Velikic G., Supic G., Maric D.L., Puletic M., Ovcak Kos M., Vojvodic D., Maric D.M. (2025). Neurodegeneration as Ecosystem Failure: A New Paradigm for Prevention and Treatment. Int. J. Mol. Sci..

[209] Qiu Y., Cheng F. (2024). Artificial intelligence for drug discovery and development in Alzheimer’s disease. Curr. Opin. Struct. Biol..

